# Balancing Accuracy and Privacy in Federated Queries of Clinical Data Repositories: Algorithm Development and Validation

**DOI:** 10.2196/18735

**Published:** 2020-11-03

**Authors:** Yun William Yu, Griffin M Weber

**Affiliations:** 1 Computer & Mathematical Sciences University of Toronto Toronto, ON Canada; 2 Department of Biomedical Informatics Harvard Medical School Boston, MA United States

**Keywords:** algorithms, medical records, privacy, information storage and retrieval, medical record linkage

## Abstract

**Background:**

Over the past decade, the emergence of several large federated clinical data networks has enabled researchers to access data on millions of patients at dozens of health care organizations. Typically, queries are broadcast to each of the sites in the network, which then return aggregate counts of the number of matching patients. However, because patients can receive care from multiple sites in the network, simply adding the numbers frequently double counts patients. Various methods such as the use of trusted third parties or secure multiparty computation have been proposed to *link* patient records across sites. However, they either have large trade-offs in accuracy and privacy or are not scalable to large networks.

**Objective:**

This study aims to enable accurate estimates of the number of patients matching a federated query while providing strong guarantees on the amount of protected medical information revealed.

**Methods:**

We introduce a novel probabilistic approach to running federated network queries. It combines an algorithm called HyperLogLog with obfuscation in the form of hashing, masking, and homomorphic encryption. It is *tunable*, in that it allows networks to balance accuracy versus privacy, and it is computationally efficient even for large networks. We built a user-friendly free open-source benchmarking platform to simulate federated queries in large hospital networks. Using this platform, we compare the accuracy, *k*-anonymity privacy risk (with *k*=10), and computational runtime of our algorithm with several existing techniques.

**Results:**

In simulated queries matching 1 to 100 million patients in a 100-hospital network, our method was significantly more accurate than adding aggregate counts while maintaining *k*-anonymity. On average, it required a total of 12 kilobytes of data to be sent to the network hub and added only 5 milliseconds to the overall federated query runtime. This was orders of magnitude better than other approaches, which guaranteed the exact answer.

**Conclusions:**

Using our method, it is possible to run highly accurate federated queries of clinical data repositories that both protect patient privacy and scale to large networks.

## Introduction

### Background

Widespread adoption of electronic health records has generated vast amounts of data, which are increasingly being used in clinical, epidemiological, and public health research [1]. Data from multiple health care organizations are often needed to increase statistical power or to access diverse patient populations and geographic regions. Although it is possible to combine patient-level data from multiple sites into a secure central repository for analysis, there are often significant technical and regulatory barriers to doing this in a way that ensures patient privacy. Institutions must compare the benefit of centralized data for research with the risk of violating the Health Insurance Portability and Accountability Act (HIPAA) and other privacy laws as a result of unintended disclosure of patient data. An alternative approach is to create federated clinical data research networks, which broadcast queries to multiple sites, run analyses locally, and then combine the results. In this way, sites retain control over their patient data. Two of the largest networks in the United States are the Patient-Centered Outcomes Research Network (PCORnet) [2] and the National Institutes of Health (NIH)–funded Accrual to Clinical Trials (ACT) network [3-5], both of which connect dozens of health care organizations across the country and include health data on nearly 100 million Americans.

As patients often receive care at more than one clinical site, the data for a patient at any one site might not be complete, and the same information about a patient might be duplicated at different sites. This can lead to queries returning incorrect results. This problem is amplified when the sites in the network are geographically close and there is greater overlap in their patient populations. However, because patients move or travel, sometimes across state or country borders, even far apart sites might share patients. A similar situation arises when patients’ data are intentionally separated for technical reasons, such as when large amounts of clinical data (eg, diagnoses and medications) and genomic data are stored in different locations, and it is not feasible to merge them into a single database. In both cases, computation must be performed in a distributed fashion, but the challenge is that an individual patient’s data may be spread across multiple databases.

**Figure 1:**
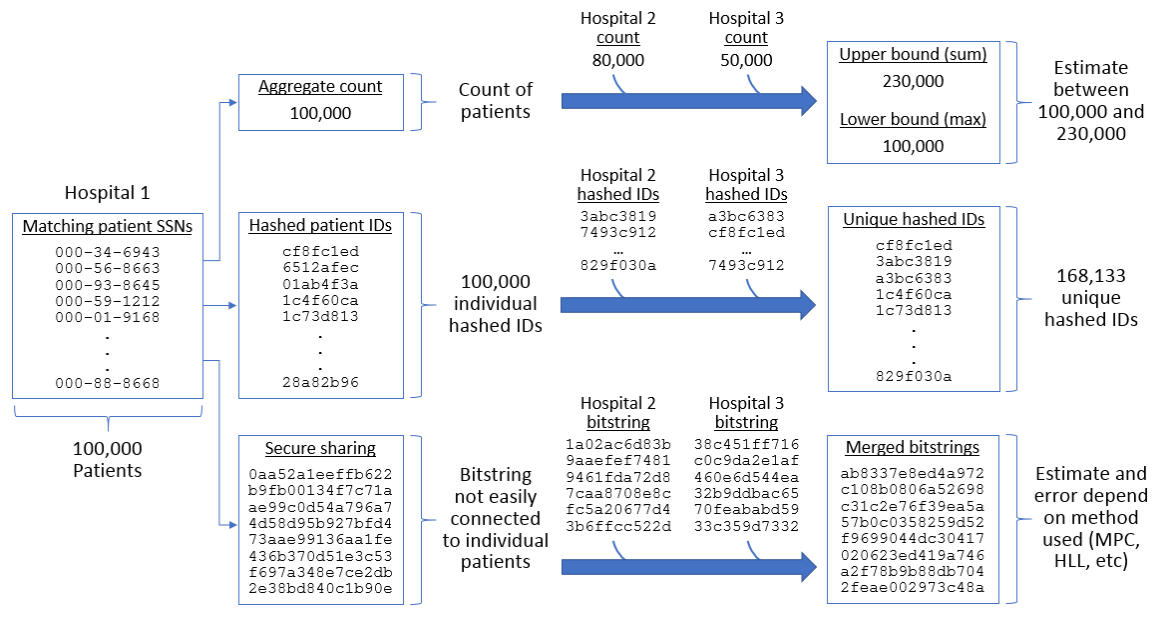
Federated query methods. We classify methods for merging distributed queries into 3 groups: (top) sharing aggregate counts, (middle) sending full hashed patient identifiers, and (bottom) generating bitstrings (displayed as hexadecimal) that do not directly correspond to individual patients but can be merged together. HLL: HyperLogLog; MPC: multiparty computation; SSN: social security number.

Various methods to addressing this problem have been described in the literature, but they have different trade-offs in terms of accuracy, privacy, scalability, and computational complexity. We grouped these into 3 broad categories: aggregate counts, hashed patient identifiers, and privacy-guaranteed methods (Figure 1).

#### Aggregate Counts

Federated queries in PCORNet and ACT ask sites to return the number of patients in their local databases who match some set of criteria, such as having both hypertension and diabetes. The networks present the user with the aggregate count from each site, and no attempt is made to link patients across sites or deduplicate records. This can lead to large overestimates of the number of distinct patients who match a query if the counts from each site are naively summed [6]. To protect patient privacy, the networks mask small counts by displaying *≤10 patients*. However, it is possible to combine results from multiple queries to reveal information about individual patients (see the *Methods* section for details). Sites participating in these networks are aware of this privacy risk, which they mitigate through institutional agreements that require sites to audit researchers’ queries and monitor their use of the network.

#### Hashed Patient Identifiers

The most accurate and semisecure method to deduplicate the results in a federated query is for each site to return the full list of patients who match the query. Privacy is the main concern, as data on every patient matching the query (potentially many millions of people) must be shared. Patient identifiers (eg, name and date of birth) [7] are typically encrypted using a one-way hash function, such as Secure Hash Algorithm 1 (SHA-1) [8]. The same patient at two sites will be hashed to the same value if the same hash function is used (and there are no inconsistencies in the underlying demographic data). Unfortunately, hash functions are vulnerable to dictionary or linkage attacks, where an adversary who knows the encryption method can simply generate a rainbow table of the hashes of many possible patient identifiers (eg, exhaustively searching all 9-digit social security numbers or accessing public voter registration lists) and then use this to reidentify the list of hash values returned by a site [9].

#### Privacy-Guaranteed Methods

Secure multiparty computation (MPC) and homomorphic encryption techniques enable true privacy guarantees in a federated network (see the *Methods* section) and have recently been introduced for distributed genome-wide association studies [10] and pharmacological collaboration [11]. The limitation of these algorithms is their computational complexity. Protocols that securely determine the number of shared patients between two sites [6,12-14] are impractical for large networks because the number of pairwise and multiway comparisons grows exponentially with the number of sites. Other approaches that avoid exponential comparison either require sharing gigabytes of data [15], making numerous rounds of back-and-forth communication [16], or using trusted third parties [17]. These are also problematic because, as we have previously shown [18], large federated clinical data networks are fragile, with multiple sites typically failing to respond even to aggregate count queries.

### HyperLogLog Sketch

In this paper, we propose a new method for combining data from sites in a federated clinical data network, based on the HyperLogLog (HLL) probabilistic sketching algorithm [19]. A probabilistic sketch is a small data structure that summarizes large amounts of data. A calculation can run on the sketch to obtain a fast, accurate estimate of what the result would be on the original data. Although HLL is widely used in many software programs, such as internet search engines, to our knowledge, it has not been applied to federated queries of health data.

The basic idea behind HLL (and other minimum value sketches) [20] is that the minimum of a collection of random numbers between 0 and 1 is inversely proportional to how many numbers are present. For example, a single random number between 0 and 1 has an expected value of 0.5; however, if we have 99 random numbers, the minimum has an expected value of 0.01. By using a hash function that maps patients to a random number between 0 and 1, we can estimate the number of patients who match a query at a site by keeping track of just the minimum hash value of the matching patients. If the minimum hash value is *v*, then the estimated number of patients is (1/*v*)-1. Although the accuracy of this estimate is poor, the method can be improved by using *t* different hash functions to generate *t* independent estimates of the number of patients. The average of these results in a more accurate overall estimate. The set of *t* minimum hash values is the sketch.

If each site in a network uses the same hash function and returns its minimal hash value, then we can estimate the number of distinct patients in the whole network that match the query from the smallest of those values. Although it may seem unintuitive that the network minimum hash is the same as the hash for one hospital, the hospital which the minimum hash corresponds to changes when multiple hash functions are used, allowing the estimator to be accurate.

Instead of using *t* hash functions, HLL improves the accuracy of this method by using a single hash function but efficiently dividing the patients into *t* partitions and returning the minimum hash value of patients in each partition. HLL also returns the position of the leading one indicator in the binary expansion of the minimum values rather than the actual values. This only has a small effect on accuracy; however, it greatly reduces the risk of reidentification from a dictionary attack. For *t* partitions, the relative error of HLL is approximately 1/sqrt(*t*). For example, by asking sites to share an HLL sketch with only 100 values, the number of distinct patients can be estimated with a 10% relative error. The error can be reduced by increasing *t*. Although *higher t* increases the risk of reidentification, the risk is quantifiable and predictable, enabling networks to define policies that maximize accuracy while reducing risk to an acceptable level.

### Objectives

We aim to enable accurate estimates of the number of unique patients matching a federated query while providing strong guarantees on the amount of protected medical information revealed.

### Structure of This Paper

In the *Methods* section, we first show how sites can generate a privacy-preserving HLL sketch of the patients who match a query and how the shared sketches from sites can be combined to estimate the number of unique patients in the network who match the query. We then describe several *obfuscation* approaches that further reduce the privacy risk of aggregate counts, hashed identifiers, and HLL sketches. These include methods that might result in a loss of information or an increase in computational complexity to make it more difficult or impossible for an adversary to identify patients. In the *Results* section, we test our algorithm and other methods using simulated networks of different sizes and degrees of patient overlap. We compare them along several dimensions, including accuracy, privacy risk, computation time, and amount of data shared. Finally, in the *Discussion* section, we summarize the trade-offs and limitations of the algorithms and provide recommendations on when networks should consider using HLL sketches.

## Methods

### Algorithms and Obfuscation Techniques for Federated Queries

Here, we describe the algorithms we compared. The basic model assumes that a researcher at one hospital in the network sends a query of the form *How many unique patients have condition X across the hospital network?* to a central network hub. The hub then distributes the query to all the hospitals in the network. The hospitals determine which of their patients match the query and return a result (the form of this result varies by algorithm) to the hub. The hub combines the results and returns an estimate of the total number of unique patients to the researcher. The name of each algorithm combines the base method (*Count*, *HashedIDs*, or *HLL*) and any additional obfuscation (*Mask*, *MPC*, *Rehash*, or *Shuffle*).

#### Count

Each hospital runs the researcher’s query locally and sends the hub a single count of the number of matching patients. The hub returns 2 numbers: (1) the maximum count from a hospital and (2) the sum of counts from all hospitals. The maximum count corresponds to a lower bound on the result, because even in the event of significant overlapping patients between hospitals, there are at least as many unique patients across the network as there are at a single hospital. For example, in Figure 1, hospitals 1, 2, and 3 have 100,000, 80,000, and 50,000 patients, respectively. It might be the case that all patients at hospitals 2 and 3 are also patients at hospital 1, which has the maximum count. However, this is not possible for the hospitals with smaller counts. For example, out of 100,000 patients of hospital 1, at most 80,000 can also be patients at hospital 2. The sum of all counts is obviously an upper bound, although it might be a substantial overestimate when there is a significant overlap between hospitals. Conversely, the maximum of all counts is obviously a lower bound.

#### Count+Mask

The procedure is identical to Count, except that if the actual count of a hospital is between 1 through 9 inclusive, the hospital returns 10 to the hub instead. This masking procedure ensures that no nonzero number corresponds to fewer than 10 patients, ensuring 10-anonymity. Both the PCORNet and ACT networks use Count+Mask. ACT further obfuscates the result by adding a small random number between –10 and +10 to the actual count [4]; however, we ignore this in our analyses.

#### Count+MPC

This protocol is based on the ElGamal cryptosystem [21] using a distributed private key to ensure that no one party can decrypt intermediate data. Only the final sum is decrypted. The individual hospital counts are hidden, even if all hospitals but one and the hub are compromised. The major disadvantage is that the MPC requires all hospitals to respond before any answer can be given. In large networks, it is likely that some hospitals will either be slow to respond or not respond at all [18], which limits this protocol to only small networks in practice (for additional information on our MPC implementation, see Multimedia Appendix 1 [6,7,10-12,14-17,21-24]).

#### HashedIDs

Each hospital runs the query locally, producing a list of matching patient IDs. Each hospital needs to use the same process for constructing IDs so that the same patient at different hospitals will have the same ID. As there is no universal patient identifier, the ID should be based on information likely to be unique to the patient and available at all hospitals, such as the concatenation of the patient’s first name, last name, and date of birth [7] (for additional details and limitations of generating a patient ID, see Multimedia Appendix 1 [6,7,10-12,14-17,21-24]). Patient IDs are encrypted using a one-way hash function. For our simulations, we used SHA-1, but in practice, a newer, more secure hash function should be used. The list of hashed IDs is then sent back to the hub. The hub then counts the number of distinct hashed IDs received from all sites and returns this as the exact answer to the query. Sites can precompute the hashed IDs for all of their patients to improve the performance of queries. Note that because *HashedIDs* uses the same hash function for all queries, a dictionary or linkage attack by the hub has a high likelihood of success.

#### HashedIDs+Rehash

This is identical to *HashedIDs*, except that the originating hospital (the hospital with the researcher who ran the query) also sends the hub a random string encrypted with the public keys of each of the other hospitals (using any kind of standard off-the-shelf asymmetric key encryption, as used in protocols such as Rivest-Shamir-Adleman [RSA] and Hypertext Transfer Protocol Secure [HTTPS]). Each hospital rehashes all the patients, prepending the random string before running it through SHA-1. By doing so, because the hub does not know the random prefix string, it cannot perform a dictionary attack to reverse the hash function, and thus, all patients get 10-anonymity. Of course, rehashing all patients with each query requires additional computational time.

#### HLL

**Figure 2:**
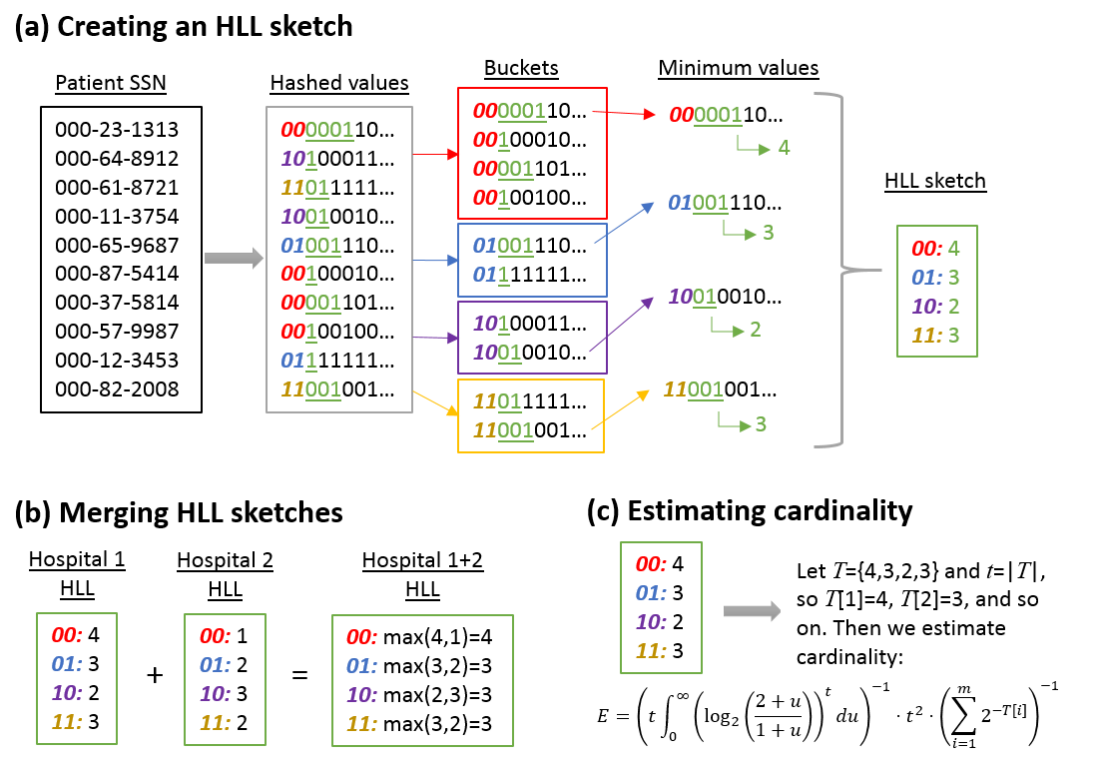
HLL sketches. (a) To create an HLL sketch, we first hash a set of identifiers for the matching patients (eg, social security number) to binary strings. The first several bits of each binary string are used to bucket the values, and then within each bucket, we store the position of the leading one indicator of the minimum value. (b) HLL sketches from different hospitals are merged by simply taking, within each bucket, the maximum value across sketches. (c) Given a list of buckets, we can estimate the cardinality. HLL: HyperLogLog; SSN: social security number.

A graphical overview of HLL is shown in Figure 2. Like HashedIDs, in HLL, the hospital uses the SHA-1 hash function to produce a 160-bit pseudorandom number for each patient that matches a query. The first 64 bits are interpreted as an integer *B*, and the patient is put into bucket *B* mod *t*, where *t* is the number of buckets. The hospital then finds the position *V* of the first bit set to 1 in bits 65 to 128 of the SHA-1 string. Within each bucket, the hospital stores the largest value *V* corresponding to a patient. The list of bucket values is the HLL sketch from that hospital. (Note that like *HashedIDs*, hospitals can precompute the buckets *B* and values *V* for all of their patients, so that this step does not have to be repeated for each query.)

The hospitals send these HLL sketches to the central hub. The hub combines the sketches by taking the maximum within each bucket across the hospital sketches, generating a sketch of the union. The hub then estimates the cardinality *C* of the union sketch using the standard HLL estimator [19]. The hub also provides a 95% CI by using the fact that the SD of the estimate is around 1/sqrt(*t*), so 1±1.96/sqrt(*t*) gives the lower and upper bounds of a 95% CI.

#### HLL+Mask

**Figure 3:**
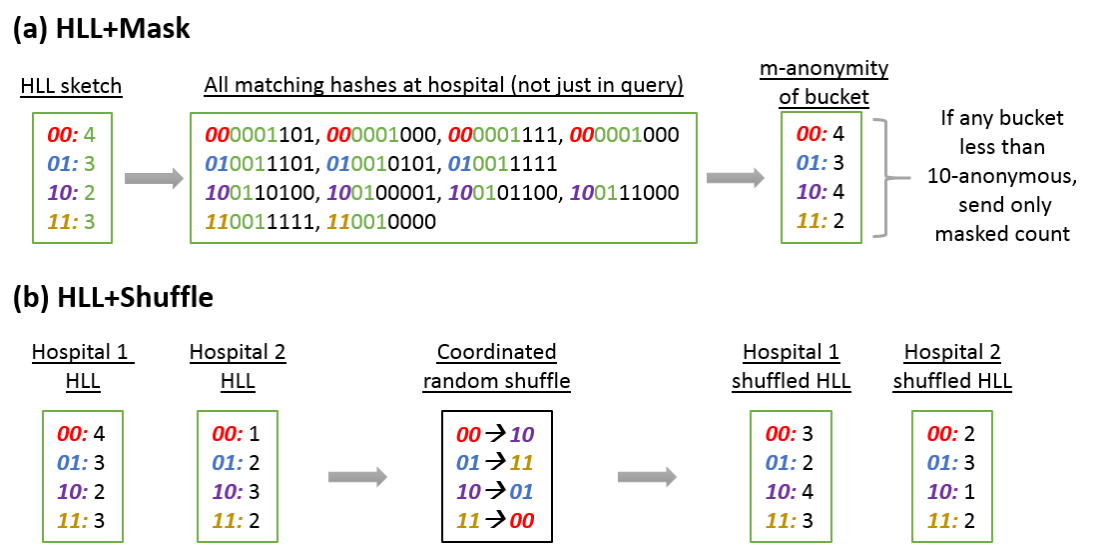
Applying obfuscation to HLL sketches. (a) HLL+Mask: For each bucket, we count the total number of patients (not just the ones who match the query) whose hashes have the same leading 1-indicator. If that number is less than 10, then the bucket is not 10-anonymous, so we do not send the HLL sketch. Instead, we only send a masked aggregate count of the number of patients matching the query. (b) HLL+Shuffle: We do a coordinated random shuffling so the central hub does not know what the original buckets were for the leading 1 indicator. However, the hub can still estimate cardinality in the same way as HLL without obfuscation. HLL: HyperLogLog.

As shown in Figure 3, this algorithm is identical to HLL, except that the hospital precomputes a list of bucket values that are less than 10-anonymous. If after generating the HLL sketch corresponding to the query, a hospital sees that there is a bucket that is not 10-anonymous, the hospital aborts and reverts to the *Count+Mask* algorithm, where only a single (possibly masked) aggregate count is returned. The hub thus receives a combination of sketches and masked counts.

The hub combines the sketches together using the HLL cardinality estimator to obtain an estimate of the count of the union of all the hospitals that sent sketches with appropriate 95% error bounds. From that, the hub goes through something similar to *Count*. The hub returns 2 numbers: the sum of all raw hospital counts plus the 95% CI maximum for the HLL union count, which gives an upper bound, and the maximum of the set of raw counts or the 95% CI minimum for the HLL union, which gives a lower bound.

#### HLL+Rehash

This algorithm uses HLL but with an obfuscation method similar to *HashedID+Rehash*. When the originating hospital sends a query to the hub, it sends both a query and a random string encrypted with public keys of each of the other hospitals in the network. The hospitals completely regenerate the HLL sketch while prepending the random string to the patient IDs before hashing. Although this procedure takes more time, the hub cannot use a dictionary attack at all because it does not know the random string. Thus, all patients are guaranteed 10-anonymity if the random string is not revealed to the hub.

#### HLL+Shuffle

This algorithm also sends a random string encrypted with public keys of each of the other hospitals in the network to the hub. However, it is much faster than *HLL+Rehash* because it avoids having to rehash all patients. Each hospital first creates an ordinary HLL sketch using their precomputed hashed IDs. It then shuffles the ordering of the buckets using the random string to determine the sort order and then sends the shuffled sketch to the hub (Figure 3).

As every hospital uses the same permutation, the sketches can still be combined and the normal estimators can be used. However, the hub, without knowing the random string, cannot know which bucket in the original sketch corresponds to a bucket in the shuffled sketch. Normally, an HLL bucket is less than 10-anonymous if that value+bucket pair corresponds to fewer than 10 individuals at the hospital. With shuffling, an HLL bucket is less than 10-anonymous only if that value corresponds to fewer than 10 individuals at the hospital. On average, this decreases the risk by dividing the risk score by the number of buckets. In other words, the buckets partition the patient population into smaller, more identifiable groups. By shuffling the buckets, it is no longer known which partition the value came from, which makes the value less identifiable.

#### HLL+MPC

Like *Count+MPC*, this method is based on the ElGamal homomorphic cryptosystem, and we use the same primitives as in that method (with the same security guarantees). We additionally take inspiration from a previous paper applying MPC to a Flajolet-Martin style approximate counter [16]. The key setup, exchange, encryption and decryption routines are identical to those of *Count+MPC* (for additional information on our MPC implementation, see Multimedia Appendix 1 [6,7,10-12,14-17,21-24]).

#### HLL+Shuffle+MPC

This procedure is simply a combination of *HLL+Shuffle* and *HLL+MPC*. Each hospital simply shuffles their buckets according to the random string before performing the encryption. The rest of the procedure is identical to that of *HLL+MPC*.

### Testing and Evaluating the Algorithms

To quantitatively measure privacy loss, we used an adapted *k*-anonymity model of privacy, whereby the privacy risk is defined to be the number of revealed data points that correspond to fewer than *k*=10 patients [22,25] (for details on the privacy risk score, see Multimedia Appendix 1 [6,7,10-12,14-17,21-24]). We ran benchmarks for runtime, accuracy, and privacy loss on (1) shared aggregate counts (*Count* and *Count+Mask*), (2) shared hashed identifiers (*HashedIDs*), and (3) our proposed HLL approach. Each of these was paired with various obfuscation techniques of masking, rehashing, shuffling, and MPC. HLL was tested using different number of buckets or values in the sketch. We indicate the size of the sketch, *t*, with a number after *HLL*, such that *HLLN* means 2^N^ values. For example, *t*=2^1^=2 (*HLL1*), *t*=2^4^=16 (*HLL4*), *t*=2^7^=128 (*HLL7*), and *t*=2^15^=32,768 (*HLL15*). Although *Count+MPC* uses a standard MPC privacy-guaranteed cryptosystem, we implemented our own protocols for the HLL+MPC variants using ElGamal encryption [21] and a private equality test [23]. We did not run benchmarks for other existing privacy-guaranteed methods because they do not scale well and are infeasible for running on large data sets, with either extremely high runtime or error (for descriptions of several of these algorithms and their limitations, see Multimedia Appendix 1 [6,7,10-12,14-17,21-24]).

Due to patient privacy, we cannot test the algorithms using actual hospital data. Therefore, we developed software for generating simulated federated networks of hospitals spread geographically with highly varying sizes and overlap [24] (for details on simulating a federated hospital network, see Multimedia Appendix 1 [6,7,10-12,14-17,21-24]). We ran our benchmarks on simulated networks containing up to 100 million total distinct patients, distributed across 100 hospitals. In the simulations, patients on average received care at 2 hospitals. However, this number varies and hospitals that are geographically close in the simulations are modeled to have a larger number of shared patients.

The benchmarks were run on an 8-core AMD Ryzen 1700 processor with 16 GB of RAM running Ubuntu 18.04.2 Long Term Support. We measured the wall-clock time for each pipeline component for time complexity and serialized bitstrings in each communication round for transmission space complexity. We provide all code in GitHub [26].

## Results

### Quantitative Simulation Benchmark Results

Multimedia Appendix 2 lists the detailed benchmark results for accuracy, privacy risk, and runtimes of queries matching 1, 10, 100, 1000, 10,000, 100,000, 1 million, 10 million, or 100 million patients using the different methods. As an example, Table 1 shows a subset of rows from the table in Multimedia Appendix 2 corresponding only to queries matching 10,000 patients and HLL sketches with 2^7^ (HLL7) and 2^15^ (HLL15) values.

Accuracy is described in absolute terms as the 95% CIs of the estimated number of patients who matched a query in 100 simulated experiments. More precisely, in each of the 100 runs, each estimator tries to return either its best guess or upper or lower bounds. If it returns a single best guess, then we report the 97.5 and 2.5 percentiles as the upper and lower bounds, respectively. If it returns upper or lower bounds, then we report the 97.5 percentile of the upper bound and the 2.5 percentile of the lower bound. These are then converted into relative errors by comparing them with the true number of distinct patients.

Privacy risk is determined by counting the number of statistics (ie, a count, HLL bucket, or hash) that are not 10-anonymous revealed to either the hub or the hub colluding with a hospital. It relates to the number of patients who are *potentially* identifiable with a specific statistic, but it does not necessarily mean that an adversary will be able to identify a patient from a statistic. Therefore, it can be thought of as an upper bound on direct linkage risk. Note that this guarantee is applicable primarily for one common threat model. In the *Discussion* section, we will cover some other more sophisticated potential avenues for attack.

Wait time is the additional computational time that hospitals require to generate the statistics plus the time the hub requires to combine each hospital’s results. (It does not include the time each hospital needs to run the query.) For the same query, hospitals might have different wait times based on the number of matching patients. We, therefore, report both *mean wait time*, which is the average hospital computation time+hub computation time, and *max wait time*, which is the maximum hospital computation time for a run+hub computation time.

**Table 1:** Benchmark results for selected methods for queries matching 10,000 patients.

Method and obfuscation		Estimated number of patients				Wait (seconds)				Risk:Hub		Risk: Hub+Site	
		Range of counts		Relative error (%)		Mean		Max					
**Count**													
	None		899.9-19,470		–91 to 95		0		0		2.65		2.65
	Mask		899.9-19,477		–91 to 95		0		0		0		0
	MPC^a^		18,886-19,470		89 to 95		0.099		0.099		0		0
**HLL7^b^**													
	None		8310-11,347		–17 to 13		0.006		0.006		15.73		15.73
	Shuffle		8310-11,347		–17 to 13		0.006		0.006		0.23		15.73
	Rehash		8310-11,347		–17 to 13		0.007		0.016		0		15.73
	Mask		7167-14,123		–28 to 41		0.005		0.005		0		0
	MPC		8310-11,347		–17 to 13		37.83		37.83		0.3		0.3
	Shuffle+MPC		8310-11,347		–17 to 13		37.83		37.83		0		0.3
**HLL15**													
	None		9928-10,075		–1 to 1		1.462		1.462		3707		3707
	Shuffle		9928-10,075		–1 to 1		1.462		1.462		0.23		3707
	Rehash		9928-10,075		–1 to 1		1.625		1.668		0		3707
	Mask		899.9-19,477		–91 to 95		0.012		0.012		0		0
**HashedIDs**													
	None		10,000-10,000		0 to 0		0.002		0.002		19,174		19,174
	Rehash		10,000-10,000		0 to 0		0.002		0.004		0		19,174

^a^MPC: multiparty computation.

^b^HLL: HyperLogLog.

As an example, in Table 1, for a query that actually matches 10,000 patients, the basic *Count* algorithm had an estimated count CI (using the summation for the upper estimate and maximum for the lower estimate) of 899.9 to 19,470 patients or a relative error of –91% to +95%. It also, on average, had 2.65 hospitals that returned potentially identifiable counts because the value was less than 10. This risk can be eliminated with *Count+Mask*, which increases the error, or by *Count+MPC*, which adds computational complexity, and only gives a single guess, instead of both upper and lower bounds. On the opposite extreme, *HashedIDs* returns the exact answer, but all 10,000 patients’ identities are at risk from a dictionary attack. (Note that Table 1 lists the risk for *HashedIDs* at 19,174 because the same patient’s hash value can be returned by more than one hospital. We report the number of potentially identifiable values shared, not the number of unique patients at risk.) In *HashedIDs+Rehash*, the hub alone cannot identify patients from the hash values (the *Risk:Hub* column). However, the risk returns if an adversary can also obtain the secret random string from a hospital (the *Risk:Hub+Site* column).

Table 1 shows that *HLL7* and *HLL15* can achieve a more tunable balance between accuracy and privacy. *HLL7* has a relative error of –17% to +13% (8310 to 11,347), which is considerably better than that of *Count*, and *HLL15* results in an even smaller relative error of –1% to 1% (9928 to 10,075). *HLL7* and *HLL15* generate, on average, 15.73 and 3707 potentially identifiable values. However, adding obfuscation with *HLL+Shuffle* adds essentially no additional computation time but reduces the risk to less than 1 (0.23 on average) potentially identifiable value. In other words, highly accurate estimates with only 1% error can be obtained with most queries having no risk of reidentification. Even if an adversary obtains the secret random string, the risk of 3707 is much less than 19,174 for *HashedIDs*.

### Graphical Comparison of Algorithms

**Figure 4:**
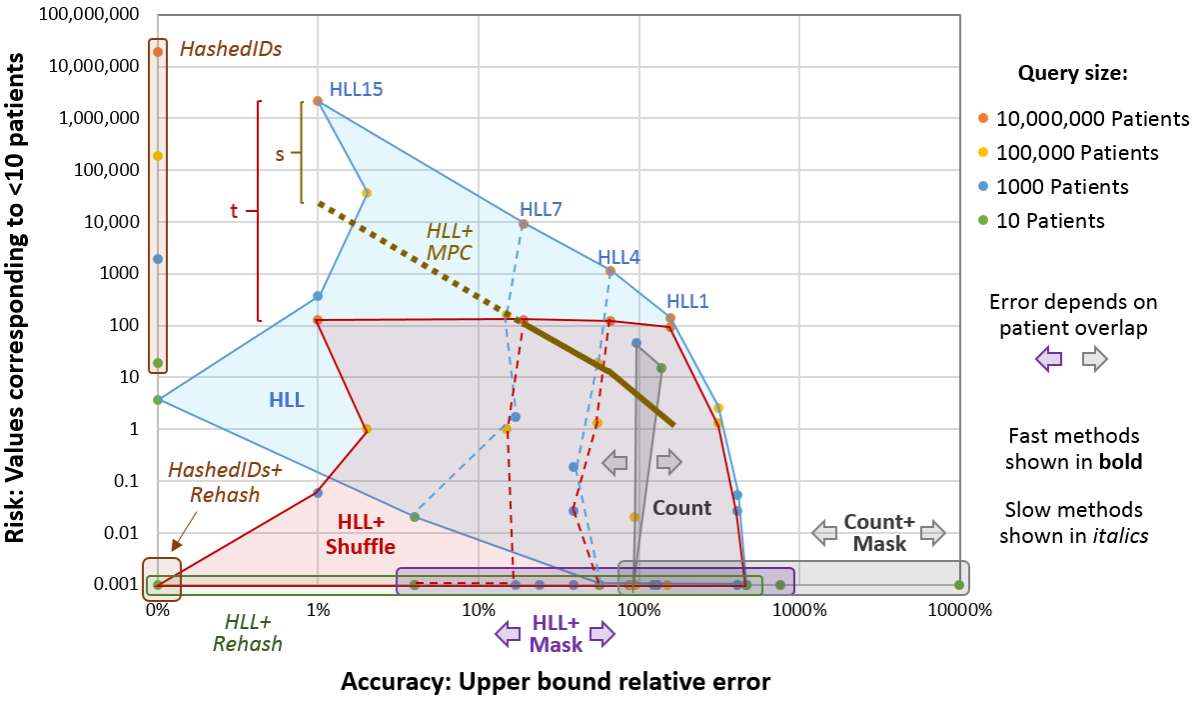
Comparison of the query accuracy/privacy risk trade-off based on the simulations of a network with 100 sites and 100 million patients. HashedIDs and Count bound the graph, whereas HLL-based methods enable a more balanced approach. (HLL+MPC is only shown for 10 million patients, and the values for HLL7+MPC and HLL15+MPC are theoretical rather than experimental.) HLL+MPC reduces the HLL risk by 1/s, where s is the number of sites in the network. HLL+Shuffle reduces the HLL risk by 1/t, where t is the number of values in the HLL sketch. HLL: HyperLogLog; MPC: multiparty computation.

Figure 4 graphically illustrates the accuracy (the horizontal axis) and risk (the vertical axis) trade-off of the different algorithms. For simplicity, only the upper bound of the relative error is used for accuracy. (The lower bound and absolute errors are not shown.) Although an individual simulation is plotted as a single point in the figure, algorithms are shown as regions because changing the input parameters to the simulation affects the results. For example, the blue region in Figure 4 covers the range of HLLs with queries of different sizes (10 to 10 million matching patients) and sketches of different sizes (HLL1=2 to HLL15=32,768 values).

The key takeaway from Figure 4 is that *Count* and *HashedIDs* are extremes that cover only one axis or the other, whereas variations of *HLL* enable networks to select an algorithm that fits anywhere between the axes. In other words, with *HLL*, networks can determine an acceptable risk level and pick the sketch size and obfuscation method that will give the most accurate result. Alternatively, they can start with a desired accuracy and pick the most secure method that runs within a given amount of time.

*Count+Mask* has the worst accuracy but guarantees 10-anonymity (thin horizontal gray box; Figure 4). As each patient in the simulation was, on average, at two hospitals, queries that matched all 100 million distinct patients returned counts from each hospital that added up to 200 million—a 100% overestimate. Queries that only matched a few patients (*small queries*) had much greater error because of the obfuscation. The worst case, in theory, is when a query matches one distinct patient and that patient happens to be at each of the 100 hospitals. As each hospital returns *≤10*, the upper bound estimate assumes that there are 10 patients in each hospital and that there is no overlap. This would result in an upper bound estimate of 100×10=1000 or a relative error of 99,900%. Even when patients are only at one hospital (no overlap), *Count+Mask* can have a 900% error.

Without obfuscation, the relative error of *Count* in the simulations remained near 100% for queries of all sizes (thin vertical gray box; Figure 4). However, for small queries, many sites returned potentially identifiable counts less than 10. At the other extreme, *HashedIDs* always gave correct answers (0% relative error). However, this requires sharing individual data on all matching patients (thin vertical brown box; Figure 4). The risk can be reduced if a different hash function is used for each query (*HashedIDs+Rehash*) and an adversary is unable to discover the hash functions.

Variations of *HLL* fill in the space between *Count*, *Count+Mash*, and *HashedIDs*, allowing the networks to tune their estimation method to achieve a more desirable balance of accuracy and risk for a given application. In Figure 4, *HLL* (the blue region), *HLL+Shuffle* (the red region), and *HLL+Rehash* (the thin horizontal green box) have the same accuracy but different levels of risk. In contrast to *Count*, which has more risk with smaller queries, *HLL*, like *HashedIDs*, has a higher risk with larger queries. Doubling the number of buckets in the *HLL* sketch reduces the error by a factor of sqrt(2); however, without obfuscation, it also doubles the risk.

The benefit of *HLL+Shuffle* is that buckets can be added to reduce error with only minimal change in risk. For queries that matched fewer than 100,000 patients, even *HLL15+Shuffle*, which has a relative error of only approximately 1%, had an average privacy risk of less than 1*. HLL+Rehash* reduced risk even further but required over a minute of extra computational time in some experiments, whereas the computational time of HLL+Shuffle is negligible. *HLL+Mask* guarantees 10-anonymity, but its error was often almost as large as *Count+Mask*. The benefit of *HLL+Mask* is that it can leverage the improved accuracy of HLL when possible, while ensuring that no added risk is introduced.

### Qualitative Comparison of the Algorithms

Table 2 provides a qualitative summary of the results. In general, HLL, especially with obfuscation, is much more accurate than aggregate counts, lower risk than sharing hash values of all matching patients, and more scalable than privacy guaranteeing algorithms. The relevant benefits of certain methods depend on the number of patients who match the query. For example, as the number of patients increases, the risk of *Count* decreases, as indicated by “(–)”, while the risk of *HLL7* increases, as indicated by “(+).”

**Table 2:** Qualitative comparison of algorithms.

Method and obfuscation		Approximation error	Runtime wait	Risk:Hub	Risk:Hub+Site
**Count**					
	None	Large	Very small	Medium (–)	Medium (–)
	Mask	Large	Very small	Zero	Zero
	MPC^a^	No change^b^	Medium	Zero	Zero
**HLL7^c^**					
	None	Medium	Small	Medium (+)	Medium (+)
	Shuffle	No change	No change	Small (+)	No change
	Rehash	No change	Medium (+)	Zero	No change
	Mask	Medium (+)	Medium (–)	Zero	Zero
	MPC	No change	Large	Small (+)	Small (+)
	Shuffle+MPC	No change	HLL7+MPC	Very small (+)	HLL7+MPC
**HLL15**					
	None	Small	Medium	Large (+)	Large (+)
	Shuffle	No change	No change	Small (+)	No change
	Rehash	No change	Medium (+)	Zero	No change
	Mask	Large (+)	Medium (–)	Zero	Zero
**HashedIDs**					
	None	Zero	Medium (+)	Very large (+)	Very large (+)
	Rehash	No change	No change	Zero	No change

^a^MPC: multiparty computation.

^b^No change: the value is the same as the method without any obfuscation.

^c^HLL: HyperLogLog.

### Computational and Communication Costs

Multimedia Appendix 3 shows the theoretical upper bounds on the computational costs of each method plus obfuscation technique, theoretical exact communication costs (the space complexity of the amount of data that the hospitals and hub have to send over the network), and the actual empirical results of both computational and communication costs.

## Discussion

### Summary of Results and Practical Considerations

In this study, we surveyed and benchmarked a range of methods for determining the number of distinct patients who matched a federated query, exploring the trade-offs in accuracy, privacy, and speed. We explicitly do not endorse a single one-size-fits-all method because different networks and institutions will have different needs. With data use agreements and a trusted third party, *HashedIDs* provides the most accurate results. When minimizing privacy risk is the most important factor, networks can choose between (1) fast but inaccurate methods such as *Count+Mask*, (2) accurate but slow algorithms such as *HLL+Rehash*, or (3) privacy-guaranteed methods that only work on small networks. A key goal of the ACT network is *real-time* queries that enable rapid exploration of the data. As a result, adding even a few seconds of computational time to ACT queries might not be acceptable. When runtimes must be minimized, methods such as *HLL7+Mask* and *HLL7+Shuffle* are fast and have a good balance between accuracy and privacy.

In practice, we envision a combination approach. Queries can first be run using a fast, private method, such as *Count+Mask* or *Count+MPC*. Given these rough results and the needs of the researcher, hospitals can then be asked to return the HLL sketches for the patients who matched the query. The initial count estimate and the privacy risk allowed by the network could be used to select the HLL sketch size and obfuscation method that would return the most accurate result in a reasonable amount of time. In the final stage of research (eg, in preparation for a full clinical trial), investigators could request permission from institutions to run accurate but potentially identifiable queries, such as *HLL15* or *HashedIDs*.

### Limitations

It is important for each institution to assess their own risk models. In particular, our risk model assumes that given a sketch for a given condition (eg, hypertension), the adversary already has access to the list of patients at the hospital and wants to identify patients that have the condition. The filled buckets of an HLL sketch correspond to hashes of patients who have the condition, and our goal is to ensure that for every patient with the condition, at least nine other patients without that condition could have hashed to the same value, ensuring 10-anonymity. Statistics that do not meet this requirement count for the privacy loss score. For example, our privacy risk analysis differs considerably from that of Desfontaines et al [27] who argue that “cardinality estimators do not preserve privacy.” However, their threat model assumes that an adversary can access the sketches as they are being generated, one patient at a time. In contrast, our risk model is based on each hospital’s final sketch, which represents all patients who match the query.

In addition, some amount of information is leaked about the patients *not* included in the sketch, precisely because they were not included. This does not allow an adversary to pinpoint patients with a condition but may sometimes allow them to determine a patient lacking that condition. Of course, this type of leakage is to some extent a problem with any aggregate query system, because if an adversary learns that only 1% of patients at a hospital have a condition, then they know with high certainty that most patients do not. In line with our analysis mentioned earlier, however, for this type of leakage, *Count* is more private than *HLL*, which is more private than *HashedIDs*, so the same privacy-accuracy trade-off applies.

We only considered a federated or distributed network in which no patient-level clinical data leave the institution and queries only return aggregate counts. This is in contrast to privacy-preserving record linkage approaches whose goal is to assemble a centralized deduplicated limited or deidentified data set through an honest broker without exchanging identifiable information. With the appropriate technologies, a secure infrastructure, and the proper institutional agreements in place, it is possible to merge data sets, even on large scales. PCORNet, in particular, has used methods similar to *HashedIDs* and *HashedIDs+Rehash* to do this for subsets of hospitals in its network [28,29]. There are multiple advantages of centralized data, including exact results and ease of use. However, in this study, we showed that (1) linking and deduplicating data at the individual patient level is not necessary to obtain accurate estimates and (2) this can be done in a computationally efficient manner. There are benefits to this federated model. It reduces concerns that hospitals might have in sharing data, it does not require updating and relinking the central database, and it places less dependency on having an honest broker.

### Conclusions

We believe that as federated data networks expand to include more institutions and data types (clinical, genomic, environmental, etc), researchers will increasingly depend on fast, accurate, and secure query tools to obtain the greatest possible scientific value from the networks. However, because no single algorithm meets all these requirements, having the ability to select among different methods for a particular application is essential. In this study, we introduce *HLL* and several obfuscation techniques to provide networks with a tunable approach to determine the number of distinct patients who match a query, which is more balanced than commonly used methods that greatly sacrifice accuracy (*Count+Mask*), privacy (*HashedIDs*), or scalability.

## Appendix

Multimedia Appendix 1 Details on the algorithms, secure methods that are not scalable to large networks, the privacy risk score, and the federated hospital network simulation.

Multimedia Appendix 2 Detailed benchmark results.

Multimedia Appendix 3 Time and space complexity for various methods.

## Abbreviations

ACT: Accrual to Clinical Trials
HLL: HyperLogLog
MPC: multiparty computation
NIH: National Institutes of Health
PCORnet: Patient-Centered Outcomes Research Network

## References

[1] Jensen PB, Jensen LJ, Brunak S (2012). Mining electronic health records: towards better research applications and clinical care. Nat Rev Genet.

[2] Fleurence RL, Curtis LH, Califf RM, Platt R, Selby JV, Brown JS (2014). Launching PCORnet, a national patient-centered clinical research network. J Am Med Inform Assoc.

[3] Weber GM, Murphy SN, McMurry AJ, Macfadden D, Nigrin DJ, Churchill S, Kohane IS (2009). The shared health research information hetwork (SHRINE): a prototype federated query tool for clinical data repositories. J Am Med Inform Assoc.

[4] McMurry AJ, Murphy SN, MacFadden D, Weber G, Simons WW, Orechia J, Bickel J, Wattanasin N, Gilbert C, Trevvett P, Churchill S, Kohane IS (2013). SHRINE: enabling nationally scalable multi-site disease studies. PLoS One.

[5] Visweswaran S, Becich MJ, D'Itri VS, Sendro ER, MacFadden D, Anderson NR, Allen KA, Ranganathan D, Murphy SN, Morrato EH, Pincus HA, Toto R, Firestein GS, Nadler LM, Reis SE (2018). Accrual to clinical trials (ACT): a clinical and translational science award consortium network. JAMIA Open.

[6] Weber GM (2013). Federated queries of clinical data repositories: the sum of the parts does not equal the whole. J Am Med Inform Assoc.

[7] Grannis SJ, Overhage JM, McDonald CJ (2002). Analysis of identifier performance using a deterministic linkage algorithm. Proc AMIA Symp.

[8] Eastlake D, Jones P US Secure Hash Algorithm 1 (SHA1). IETF Tools.

[9] Oechslin P (2003). Making a Faster Cryptanalytic Time-memory Trade-Off. Annual International Cryptology Conference.

[10] Cho H, Wu DJ, Berger B (2018). Secure genome-wide association analysis using multiparty computation. Nat Biotechnol.

[11] Hie B, Cho H, Berger B (2018). Realizing private and practical pharmacological collaboration. Science.

[12] Kolesnikov V, Matania N, Pinkas B, Rosulek M, Trieu N (2017). Practical Multi-Party Private Set Intersection From Symmetric-Key Techniques. Proceedings of the 2017 ACM SIGSAC Conference on Computer and Communications Security.

[13] de Cristofaro CE, Gasti P, Tsudik G (2012). Fast and Private Computation of Cardinality of Set Intersection and Union. International Conference on Cryptology and Network Security.

[14] Swamidass SJ, Matlock M, Rozenblit L (2015). Securely measuring the overlap between private datasets with cryptosets. PLoS One.

[15] Fenske E, Mani A, Johnson A, Sherr M (2017). Distributed Measurement With Private Set-Union Cardinality. Proceedings of the 2017 ACM SIGSAC Conference on Computer and Communications Security.

[16] Dong C, Loukides G (2017). Approximating private set union/intersection cardinality with logarithmic complexity. IEEE Trans Inform Forensic Secur.

[17] Yigzaw KY, Michalas A, Bellika JG (2017). Secure and scalable deduplication of horizontally partitioned health data for privacy-preserving distributed statistical computation. BMC Med Inform Decis Mak.

[18] Weber GM (2015). Federated queries of clinical data repositories: scaling to a national network. J Biomed Inform.

[19] Flajolet P, Fusy E, Gandouet O, Meunier F (2007). Hyperloglog: the Analysis of a Near-Optimal Cardinality Estimation Algorithm. Conference on Analysis of Algorithms.

[20] Bar-Yossef Z, Jayram T, Kumar R, Sivakumar D, Trevisan L (2002). Counting Distinct Elements in a Data Stream. International Workshop on Randomization and Approximation Techniques in Computer Science.

[21] Elgamal T (1985). A public key cryptosystem and a signature scheme based on discrete logarithms. IEEE Trans Inform Theory.

[22] El Emam K, Dankar F (2008). Protecting privacy using k-anonymity. J Am Med Inform Assoc.

[23] Jakobsson M, Juels A (2000). Mix and Match: Secure Function Evaluation via Ciphertexts. International Conference on the Theory and Application of Cryptology and Information Security.

[24] Berry BJ (1961). City size distributions and economic development. Econ Dev Cult Change.

[25] Sweeney L (2012). K-anonymity: a model for protecting privacy. Int J Unc Fuzz Knowl Based Syst.

[26] yunwilliamyu / secure-distributed-union-cardinality. GitHub.

[27] Desfontaines D, Lochbihler A, Basin D (2020). Cardinality estimators do not preserve privacy. ArXiv.

[28] Kho AN, Cashy JP, Jackson KL, Pah AR, Goel S, Boehnke J, Humphries JE, Kominers SD, Hota BN, Sims SA, Malin BA, French DD, Walunas TL, Meltzer DO, Kaleba EO, Jones RC, Galanter WL (2015). Design and implementation of a privacy preserving electronic health record linkage tool in Chicago. J Am Med Inform Assoc.

[29] Bian J, Loiacono A, Sura A, Mendoza VT, Lipori G, Guo Y, Shenkman E, Hogan W (2019). Implementing a hash-based privacy-preserving record linkage tool in the oneFlorida clinical research network. JAMIA Open Sep.

