# Supplementary material for: Balancing Accuracy and Privacy in Federated Queries of Clinical Data Repositories: Algorithm Development and Validation
Source: J Med Internet Res. 2020 Nov 3;22(11):e18735. doi: 10.2196/18735 (PMC7671849; doi:10.2196/18735)
Supplement: Multimedia Appendix 1 [file jmir_v22i11e18735_app1.pdf]

Multimedia Appendix 1

Federated queries of clinical data repositories: balancing accuracy and privacy

Yun William Yu, PhD
Computer and Mathematical Sciences
University of Toronto at Scarborough
Toronto, ON M5S 1J7
ywyu@math.toronto.edu

Griffin M Weber, MD, PhD
Department of Biomedical Informatics
Harvard Medical School
Boston, MA 02115
weber@hms.harvard.edu

Table of Contents

1) Secure multi-party computation (MPC) algorithm details 3
2) Generating patient IDs for hash functions 6
3) Secure methods that are not scalable to large networks 7
4) Privacy risk score (m-anonymity) 9
5) Simulating a federated hospital network 10

Secure multi-party computation (MPC) algorithm details

Below are implementation details of our *Count+MPC* and *HLL+MPC* algorithms.

Count+MPC

This protocol is based on the ElGamal cryptosystem [ElGamal 1985] using a distributed private key to ensure that no one party can decrypt intermediate data, secure in the Honest But Curious even if all hospitals but one and the hub are compromised. We take advantage of the multiplicatively homomorphic property of ElGamal encryption, which means that given an encryption function E and decryption function D, $D\big(E(ab)\big) = ab$. It is secure in the semi-honest framework assuming the difficult of the discrete logarithm problem. The algorithm has three parts (key generation, encryption, and decryption), which requires two rounds of communication between the hospitals and the hub.

Key generation.
We use a fixed 1024-bit prime p and appropriate generator g as the basis of all our cryptographic keys (see Supplementary Information: ElGamal constants). All arithmetic will be performed in that prime field defined by p (i.e. will be performed modulus p). Given p and g, a private/public keypair consists of a random number $x \in [2, p-1]$ as the private key, and $y = g^x$. We wish to ensure that no one party ever has access to x, so we use a distributed key generation protocol. Each hospital i generates a random x_i and produces the corresponding $y_i = g^{x_i}$, which it sends to the central hub. The hub then computes $y = \prod y_i$. Note that $y = \prod g^{x_i} = g^{\sum x_i}$, so y is the public key corresponding to the private key $x = \sum x_i$, but no hospital actually knows the summed value x. The hub returns the public key y to each hospital.

Encryption.
The standard ElGamal encryption function $E(m) = (g^z, my^z)$, where z in a random integer in the field. Note that ElGamal has the nice property that the same plaintext message will be encrypted to a many possible encrypted ciphertexts because of z. This is essential to defeating dictionary attacks on the ciphertext. As a technical note, in order to have provable security, the message m must be a quadratic residue of the field, which we will ensure the in the protocol described.
<u>Decryption</u>. The standard ElGamal decryption function $D\big((c_1, c_2)\big) = (c_1^x)^{p-2} \cdot c_2$. Note that we can do a distributed decryption for a ciphertext by sending each hospital c_1, and asking the hospitals to return $c_1^{x_i}$, which when multiplied together give c_1^x.

Round 1: encryption and summation.
Each hospital runs the query locally, producing a count a_i, and then sends the value $E(4^{a_i})$ back to the hub (we use 4^{a_i} because it is a quadratic residue). The hub computes $\sum E(4^{a_i}) = E\big(4^{\sum a_i}\big)$.

Round 2: decryption.
The encrypted sum is decrypted using the distributed decryption protocol described above, giving $4^{\sum a_i}$. Of course, performing discrete logarithms is hard (or else ElGamal encryption would not be secure), but we can precompute a discrete log table for powers

of 4 relatively easily. Using that lookup table, the hub produces $\sum a_i$ as the final response for the query.

HLL+MPC

Like Count + MPC, this method is based off of the ElGamal homomorphic cryptosystem, and we use the same primitives as in that method (with the same security guarantees). We additionally take inspiration from a previous paper applying MPC to a Flajolet-Martin style approximate counter [Dong 2017]. The key setup and exchange are identical to Count + MPC, as well as the encryption and decryption routines, so we only describe the following rounds:

Round 1: encryption and merging.
Each hospital begins by generating an HLL sketch of the query. We then unroll each bucket $B_j = v_j$ of the sketch into a binary string of length 32 with v_j 1's and $32 - v_j$ 0's. i.e. if $v_j = 10$, the binary string would be "11111111110000000000000000000000". However, ElGamal homomorphic encryption is only secure when using non-zero quadratic residues of the prime field. So we turn that string into a vector, replacing 1's with 4's and 0's with 1's, resulting in a vector of length 32, [4, 4, 4, 4, 4, 4, 4, 4, 4, 4, 1, …, 1]. The hospital then encrypts each of these unrolled bucket vectors into $[E(4), \ldots, E(4), E(1), \ldots E(1)]$, and send them to the hub. Note that we rely the fact that ElGamal encryption is probabilistic, so each of the 4's encrypts to a different ciphertext, and so do each of the 1's. Thus, the encrypted vector does not reveal any information about the underlying binary bitstring.

The hub receives the encrypted HLL sketches from each hospital, and then takes the product across hospitals of each position in the unrolled bucket vectors, giving a product vector $\left[\prod x_{1,i}, \ldots, \prod x_{32,i}\right]$. Because ElGamal is multiplicatively homomorphic, $\prod x_{1,i} = E(1)$ if and only if all $x_{j,i} = E(1)$. Were we to decrypt this vector, it would reveal the maximum bucket value for this bucket, because the vector would be equal to 1 at all indices above that value. However, this leaks information because the other indices would have some value 4^y, where y is the number of times a hospital had a value of at least that index.

To resolve this information leakage, we use a private equality test [Jakobsson 2000]. Given two ciphertexts (c_1, c_2) and (c_1', c_2'), $T = \left(\left(\frac{c_1'}{c_1}\right)^z, \left(\frac{c_2'}{c_2}\right)^z\right) = \left(\left(c_1' \cdot c_1^{p-2}\right)^z, \left(c_2' \cdot c_2^{p-2}\right)^z\right)$, where z is a random integer, is a private equality test. More precisely, $D(T) = 1$ if and only if $D\big((c_1, c_2)\big) = D\big((c_1', c_2')\big)$. More importantly, $D(T)$ is a random integer (different from z) if the two ciphertexts were not equal in the plaintext space. The hub thus does a private equality test of all the combined encrypted bucket values, testing if they are equal to 1, and masking the result if they are not equal to 1. Those new masked vectors do not leak any information, revealing only the maximum value of the bucket across hospitals.

Round 2: decryption.
We now run the distributed decryption protocol on each of those masked vector elements. Because each element is independent, they can be decrypted in parallel in only one round of communication. For each bucket, the hub then looks at the maximum index that is not equal to 1, which corresponds to the maximum bucket value across

hospitals; this procedure allows the hub to reconstruct the merged HLL sketch. Once given a merged HLL sketch, the hub can then follow the rest of the standard procedure for the HyperLogLog method.

ElGamal constants

The 1024-bit prime we used is:

```
98028036272659031371560742242955169861910550634811171303401
```

The generator we used is:

```
24206408586964102421812765517240973131319511671044202856202
```

Note that while we have attempted to choose appropriate constants, we are not cryptographers. As with any secure system, we recommend that any real-world implementation be audited by a security professional. To wit, a real-world deployment would probably implement ElGamal over Curve25519 using the standard generators, and be implemented using a hardened crypto library like LibSodium. However, that is beyond the scope of this work.

Generating patient IDs for hash functions

For both the HyperLogLog and HashedIDs methods, a unique identifier (ID) is needed for each patient. Each hospital needs to use the same ID for the same patient. Because there is no universal patient identifier (UPI), the ID should be based on information likely to be unique to the patient and available at all hospitals, such as the concatenation of the patient's first name, last name, and date of birth. However, there are limitations to this because of missing data, data errors (e.g., a misspelled name), and data that change over time (e.g., a person changes his or her name).

The best combination of demographic variables depends on whether it is preferable for queries to over- or under-count the number of matching patients [Grannis SJ 2002]. For example, using just first initial plus last name will cause many people to have the same ID; while, combining first and last name, date of birth, social security number, and zip code increases the chance that the same person has different IDs at different hospitals.

Although it would increase query time and privacy risk, a hospital network can generate multiple IDs for each patient, using different combinations of variables. The same query can be run once for each ID, producing a range of results. The researcher can use this to gauge what the true number of matching patients might be.

Secure methods that are not scalable to large networks

We quantitatively benchmarked two secure MPC protocols. *Count+MPC* is just a straight-forward implementation of secure MPC summation, but *HLL+MPC* is a protocol we developed ourselves, inspired by Dong, et al [Dong 2017]. The reason we developed that protocol instead of using existing protocols from the cryptographic literature is that most such methods are impractically slow, due to bad scaling of communication and computation requirements. Here, we describe a few secure MPC protocols that provide privacy guarantees without the need for a trusted 3rd party. However, because secure MPC and homomorphic encryption are computationally complex, they could take on the order of days to weeks for a single query in a large network. This makes them impractical except for very small networks. As a result, we do not include them in our benchmarking simulations.

One MPC approach is to use a pairwise private intersection protocol [Kolesnikov 2017, de Christofaro 2012], which securely determines the number of shared patients between two sites. Subtracting this from the sum of the counts from each site gives the total number of distinct patients. However, the number of required pairwise and multi-way comparisons grows exponentially with the number of sites, making this impractical for large networks. Patient partitioning [Weber 2013] and cryptosets [Swamidass 2015] are related non-MPC methods that have similar scalability problems due to the number of patient slices. A recent approach using counting Bloom filters is able to solve the deduplication problem without pairwise comparisons, but due to the nature of Bloom filters scales linearly in the number of patients and requires at least two trusted data custodians even in a semi-honest framework [Yigzaw 2017].

Other work in the MPC literature has produced algorithms for directly computing unions and deduplications of sets without the problem of exponential comparisons. Unfortunately, this comes at the cost of either significantly more computation time and communication bandwidth requirements, which can be on the order of gigabytes of shared data for a single query, with linear communication complexity and super-linear time-complexity in the number of patients [Fenske 2017]. A more recent approach combines a Flajolet-Martin style estimator with a secure MPC protocol [Dong 2017]. The algorithm has logarithmic space complexity, in that the number of bits needed scales logarithmically with respect to the number of patients who match the query. However, the trade-off is that it requires numerous back-and-forth communication---on the order of $\log N$ rounds, where N is the total patient population---between all the hospitals in the network to execute the protocol. As mentioned above though, our HLL + MPC protocol is heavily based off of Dong, et al.

The root of the issue is that in the context of a federated network of hospitals, if each hospital acts as a computing party for an MPC protocol, then each hospital can guarantee to itself that at least it itself is not malicious. This feature is desirable for hospitals, because it means they do not have to trust anyone but themselves. However, most MPC methods scale badly in the number of computing parties; using semi-trusted dedicated compute parties can help, but that still requires trusting those compute parties to not collude. In recent years, more scalable secure MPC protocols have been introduced to solving distributed genome-wide association studies [Cho 2018] and pharmacological collaborations [Hie 2018], but these protocols are not practical for the

near-real-time results that clinical researchers expect (indeed, in that context, it is considered fast to get results in weeks). For this reason, we only compared against the two MPC protocols we ourselves implemented, which are designed to be scalable at the level we need for clinical queries.

Privacy risk score (k-anonymity)

We define a piece of aggregate information, or statistic, as less than k-anonymous if it includes at least 1 individual and could have been generated by fewer than k individuals in some background population. As long as patients have 2-anonymity, they have not been fully revealed. However, in practice, hospitals are usually more conservative. One study recommended 5-anonymity for hospitals [Emam 2008], but the national PCORNet and ACT networks go even higher, requiring 10-anonymity. For purposes of this paper, we will use 10-anonymity throughout our analysis to be consistent with these existing networks. We will define the privacy risk for a release of data as the number of statistics revealed to the adversary that are not 10-anonymous.

For the background population we use the patient population at a hospital, because the hub generally knows when a piece of information comes from a particular hospital. In the case where we use MPC to merge data across the network, however, the background population can be taken to be the patient population across the entire network, as no one party sees the information from a single hospital.

In the case of a single count from a hospital, whether or not that count is 10-anonymous is easy to determine: if the count is between 1 and 9 inclusive, then it is not 10-anonymous; else, it is. Note that this is not a perfect proxy, because while a single count may be 10-anonymous, multiple counts from the same hospital might not be. For example, if the count of male patients is 10 and the count of male + female patients is 11, then two counts, while individually 10-anonymous, can together be combined to reveal that there is only 1 female patient. Although here we analyze only the privacy risk from revealing a single count from a hospital, so we do not worry about that, it is still worth remembering that even aggregate counts >10 are not perfect 10-anonymity.

For a hashed value generated from a patient ID, we consider it 10-anonymous if the adversary cannot reverse the hash function to figure out the original patient ID to within 10 patients. Luckily, cryptographic hash functions are one-way, meaning that the function cannot be directly reversed. Unfortunately, since the space of patient IDs (e.g. social security numbers) is constrained, an adversary can simply create a rainbow table of the hashed values of every possible patient ID, and then simply do a lookup. Thus, a hashed value is only 10-anonymous if at least 10 patients in the background population hash to that particular value. Unfortunately, for hashed IDs that are sufficiently large to do deduplication of patients (e.g. 32- or 64-bits), the very property that allows deduplication also ensures that close to none of the hashed IDs are 10-anonymous.

HyperLogLog buckets can be thought of as a much shorter hashed ID. Whereas we might use a 64-bit hash when using Hashed IDs, the HyperLogLog bucket stores only the position of the first 1 bit in that 64-bit hash. This increases the number of collisions considerably. An HLL bucket with value x is 10-anonymous if at least 10 patients in the background population have hashes where the leading 1-indicator indicator is in position x, which happens much more often. Additionally, there are generally many fewer HLL buckets than patient IDs, so fewer potentially risky statistics are revealed to begin with.

Simulating a federated hospital network

Because of patient privacy, we cannot test the algorithms using actual hospital data. We therefore built a simulation of a set of hospitals spread geographically with highly varying sizes and overlap.

First we model geographic spread by placing 100 cities uniformly randomly in a 2D unit square. City sizes are often modeled to have lognormal distributions [Berry 1961], with probability density function

$$p(x) = \frac{1}{\sigma x\sqrt{2\pi}} \exp\left(-\frac{(\ln x - \mu)^2}{2\sigma^2}\right),$$

where μ and σ are respectively the mean and standard deviation of the underlying normal distribution.

Each of the 100 hospitals in our network is assumed to draw primarily from one of those cities. We randomly sample 100 numbers from a lognormal distribution with $\mu = 0$ and $\sigma = 1.2$, and then scale up all the numbers such that the sum is 100 million total unique patients. Each patient is assigned a number between 1 and 100 million, and is then placed in one of the 100 hospitals as their home hospital, according to the scaled up lognormal size distribution computed earlier.

For each patient, we draw a random integer from a binomial distribution $B\left(9, \frac{1}{9}\right)$, which will denote the number of additional hospitals patients are assigned to. Here the intuition is that most patients are only at a single hospital, but some patients are admitted to many hospitals. However, by choosing those parameters of the binomial distribution, we ensure that on average, patients are admitted to 2 hospitals (their home hospital, and one additional one as the mean of the binomial distribution is 1).

Then we assume that patients who are admitted to multiple hospitals are more likely to go to nearby ones, according to the hospital locations in the unit square we assigned earlier. We assume that the probability that a patient chooses a particular additional hospital is inversely proportional to the square of the distance between the new hospital and the patient's home hospital. Using this probability distribution, we assign each patient to their additional hospitals.

By using this procedure, we generate hospitals that start with lognormal sizes, following city size distributions, but with some smoothing of the sizes because some patients will go to multiple hospitals.
