# Supplementary material for: Balancing Accuracy and Privacy in Federated Queries of Clinical Data Repositories: Algorithm Development and Validation
Source: J Med Internet Res. 2020 Nov 3;22(11):e18735. doi: 10.2196/18735 (PMC7671849; doi:10.2196/18735)
Supplement: Multimedia Appendix 2 [file jmir_v22i11e18735_app2.pdf]

Multimedia Appendix 2

Federated queries of clinical data repositories: balancing accuracy and privacy

Yun William Yu, PhD
Computer and Mathematical Sciences
University of Toronto at Scarborough
Toronto, ON M5S 1J7
ywyu@math.toronto.edu

Griffin M Weber, MD, PhD
Department of Biomedical Informatics
Harvard Medical School
Boston, MA 02115
weber@hms.harvard.edu

Table A1. Detailed benchmark results

We measure the accuracy, wait time (in seconds), and privacy risk of running the query using raw counts, HyperLogLog sketches of various sizes, and sending full Hashed patient IDs.

Table A1a. 1 patient query

Method	Obfuscation	Estimated number of patients		Wait (s)		Risk: Hub	Risk: Hub+Site
		Range of Counts	Relative Error	Mean	Max		
Count	None	[1.000, 3.525]	[0%, 252%]	0	0	1.91	1.91
	Mask	[10.000, 35.25]	[900%, 3425%]	0	0	0	0
	MPC	[1.000, 3.525]	[0%, 252%]	0.099	0.099	0	0
HLL0	None	[0.694, 22.20]	[-31%, 2120%]	0	0	0	0
	Shuffle	[0.694, 22.20]	[-31%, 2120%]	0	0	0	0
	Rehash	[0.694, 22.20]	[-31%, 2120%]	0	0	0	0
	Mask	[0.694, 22.20]	[-31%, 2120%]	0	0	0	0
	MPC	[0.694, 22.20]	[-31%, 2120%]	0.385	0.385	0	0
	Shuffle+MPC	[0.694, 22.20]	[-31%, 2120%]	0.385	0.385	0	0
HLL1	None	[1.386, 1.386]	[39%, 39%]	0	0	0	0
	Shuffle	[1.386, 1.386]	[39%, 39%]	0	0	0	0
	Rehash	[1.386, 1.386]	[39%, 39%]	0	0	0	0
	Mask	[1.386, 1.386]	[39%, 39%]	0	0	0	0
	MPC	[1.386, 1.386]	[39%, 39%]	0.675	0.675	0	0
	Shuffle+MPC	[1.386, 1.386]	[39%, 39%]	0.675	0.675	0	0
HLL4	None	[1.033, 1.033]	[3%, 3%]	0.001	0.001	0	0
	Shuffle	[1.033, 1.033]	[3%, 3%]	0.001	0.001	0	0
	Rehash	[1.033, 1.033]	[3%, 3%]	0.001	0.001	0	0
	Mask	[1.033, 1.033]	[3%, 3%]	0.001	0.001	0	0
	MPC	[1.033, 1.033]	[3%, 3%]	4.781	4.781	0	0
	Shuffle+MPC	[1.033, 1.033]	[3%, 3%]	4.781	4.781	0	0
HLL7	None	[1.004, 1.004]	[0%, 0%]	0.006	0.006	0	0
	Shuffle	[1.004, 1.004]	[0%, 0%]	0.006	0.006	0	0
	Rehash	[1.004, 1.004]	[0%, 0%]	0.007	0.007	0	0
	Mask	[1.004, 1.004]	[0%, 0%]	0.007	0.007	0	0
	MPC	[1.004, 1.004]	[0%, 0%]	37.73	37.73	0	0
	Shuffle+MPC	[1.004, 1.004]	[0%, 0%]	37.73	37.73	0	0
HLL15	None	[1.000, 1.000]	[0%, 0%]	1.463	1.463	0.31	0.31
	Shuffle	[1.000, 1.000]	[0%, 0%]	1.463	1.463	0	0.31
	Rehash	[1.000, 1.000]	[0%, 0%]	1.623	1.647	0	0.31
	Mask	[1.000, 21.00]	[0%, 2000%]	1.632	1.632	0	0
Hashed IDs	None	[1.000, 1.000]	[0%, 0%]	0.001	0.001	1.91	1.91
	Rehash	[1.000, 1.000]	[0%, 0%]	0.001	0.001	0	1.91

Table A1b. 10 patient query.

Method	Obfuscation	Estimated number of patients		Wait (s)		Risk: Hub	Risk: Hub+Site
		Range of Counts	Relative Error	Mean	Max		
Count	None	[1.475, 24.00]	[-85%, 140%]	0	0	15.72	15.72
	Mask	[10.000, 205.2]	[0%, 1952%]	0	0	0	0
	MPC	[15.47, 24.00]	[55%, 140%]	0.099	0.099	0	0
HLL0	None	[2.776, 88.82]	[-72%, 788%]	0	0	0	0
	Shuffle	[2.776, 88.82]	[-72%, 788%]	0	0	0	0
	Rehash	[2.776, 88.82]	[-72%, 788%]	0	0	0	0
	Mask	[2.776, 88.82]	[-72%, 788%]	0	0	0	0
	MPC	[2.776, 88.82]	[-72%, 788%]	0.385	0.385	0	0
	Shuffle+MPC	[2.776, 88.82]	[-72%, 788%]	0.385	0.385	0	0
HLL1	None	[3.332, 35.23]	[-67%, 252%]	0	0	0	0
	Shuffle	[3.332, 35.23]	[-67%, 252%]	0	0	0	0
	Rehash	[3.332, 35.23]	[-67%, 252%]	0	0	0	0
	Mask	[3.332, 35.23]	[-67%, 252%]	0	0	0	0
	MPC	[3.332, 35.23]	[-67%, 252%]	0.675	0.675	0	0
	Shuffle+MPC	[3.332, 35.23]	[-67%, 252%]	0.675	0.675	0	0
HLL4	None	[5.995, 15.69]	[-40%, 57%]	0.001	0.001	0	0
	Shuffle	[5.995, 15.69]	[-40%, 57%]	0.001	0.001	0	0
	Rehash	[5.995, 15.69]	[-40%, 57%]	0.001	0.001	0	0
	Mask	[5.995, 15.69]	[-40%, 57%]	0.001	0.001	0	0
	MPC	[5.995, 15.69]	[-40%, 57%]	4.778	4.778	0	0
	Shuffle+MPC	[5.995, 15.69]	[-40%, 57%]	4.778	4.778	0	0
HLL7	None	[8.261, 10.41]	[-17%, 4%]	0.006	0.006	0.01	0.01
	Shuffle	[8.261, 10.41]	[-17%, 4%]	0.006	0.006	0	0.01
	Rehash	[8.261, 10.41]	[-17%, 4%]	0.007	0.007	0	0
	Mask	[8.261, 10.41]	[-17%, 4%]	0.007	0.007	0	0
	MPC	[8.261, 10.41]	[-17%, 4%]	37.72	37.72	0	0
	Shuffle+MPC	[8.261, 10.41]	[-17%, 4%]	37.72	37.72	0	0
HLL15	None	[10.00, 10.00]	[0%, 0%]	1.477	1.477	4	4
	Shuffle	[10.00, 10.00]	[0%, 0%]	1.477	1.477	0	4
	Rehash	[10.00, 10.00]	[0%, 0%]	1.636	1.659	0	3.8
	Mask	[10.000, 87.53]	[0%, 775%]	1.578	1.578	0	0
Hashed IDs	None	[10.000, 10.000]	[0%, 0%]	0.002	0.002	19	19
	Rehash	[10.000, 10.000]	[0%, 0%]	0.002	0.002	0	19

Table A1c. 100 patient query.

Method	Obfuscation	Estimated number of patients		Wait (s)		Risk: Hub	Risk: Hub+Site
		Range of Counts	Relative Error	Mean	Max		
Count	None	[9.000, 205.6]	[-91%, 106%]	0	0	59.78	59.78
	Mask	[10.000, 734.1]	[-90%, 634%]	0	0	0	0
	MPC	[176.9, 205.6]	[77%, 106%]	0.099	0.099	0	0
HLL0	None	[11.10, 541.8]	[-89%, 442%]	0	0	0	0
	Shuffle	[11.10, 541.8]	[-89%, 442%]	0	0	0	0
	Rehash	[11.10, 541.8]	[-89%, 442%]	0	0	0	0
	Mask	[11.10, 541.8]	[-89%, 442%]	0	0	0	0
	MPC	[11.10, 541.8]	[-89%, 442%]	0.384	0.384	0	0
	Shuffle+MPC	[11.10, 541.8]	[-89%, 442%]	0.384	0.384	0	0
HLL1	None	[19.99, 807.6]	[-80%, 708%]	0	0	0	0
	Shuffle	[19.99, 807.6]	[-80%, 708%]	0	0	0	0
	Rehash	[19.99, 807.6]	[-80%, 708%]	0	0	0	0
	Mask	[19.99, 807.6]	[-80%, 708%]	0	0	0	0
	MPC	[19.99, 807.6]	[-80%, 708%]	0.676	0.676	0	0
	Shuffle+MPC	[19.99, 807.6]	[-80%, 708%]	0.676	0.676	0	0
HLL4	None	[58.08, 158.6]	[-42%, 59%]	0.001	0.001	0	0
	Shuffle	[58.08, 158.6]	[-42%, 59%]	0.001	0.001	0	0
	Rehash	[58.08, 158.6]	[-42%, 59%]	0.001	0.001	0	0
	Mask	[58.08, 158.6]	[-42%, 59%]	0.001	0.001	0	0
	MPC	[58.08, 158.6]	[-42%, 59%]	4.782	4.782	0	0
	Shuffle+MPC	[58.08, 158.6]	[-42%, 59%]	4.782	4.782	0	0
HLL7	None	[87.68, 115.3]	[-12%, 15%]	0.006	0.006	0.07	0.07
	Shuffle	[87.68, 115.3]	[-12%, 15%]	0.006	0.006	0	0.07
	Rehash	[87.68, 115.3]	[-12%, 15%]	0.007	0.007	0	0.07
	Mask	[87.68, 118.0]	[-12%, 18%]	0.007	0.007	0	0
	MPC	[87.68, 115.3]	[-12%, 15%]	37.65	37.65	0	0
	Shuffle+MPC	[87.68, 115.3]	[-12%, 15%]	37.65	37.65	0	0
HLL15	None	[99.15, 100.2]	[-1%, 0%]	1.44	1.44	36.12	36.12
	Shuffle	[99.15, 100.2]	[-1%, 0%]	1.44	1.44	0	36.12
	Rehash	[99.15, 100.2]	[-1%, 0%]	1.6	1.625	0	36.12
	Mask	[48.41, 464.9]	[-52%, 365%]	1.207	1.207	0	0
Hashed IDs	None	[100.00, 100.00]	[0%, 0%]	0.002	0.002	190.6	190.6
	Rehash	[100.00, 100.00]	[0%, 0%]	0.002	0.002	0	190.6

Table A1d. 1,000 patient query.

Method	Obfuscation	Estimated number of patients		Wait (s)		Risk: Hub	Risk: Hub+Site
		Range of Counts	Relative Error	Mean	Max		
Count	None	[93.47, 1,973]	[-91%, 97%]	0	0	43.84	44
	Mask	[93.47, 2,236]	[-91%, 124%]	0	0	0	0
	MPC	[1,863, 1,973]	[86%, 97%]	0.099	0.099	0	0
HLL0	None	[177.6, 22,737]	[-82%, 2174%]	0	0	0	0
	Shuffle	[177.6, 22,737]	[-82%, 2174%]	0	0	0	0
	Rehash	[177.6, 22,737]	[-82%, 2174%]	0	0.001	0	0
	Mask	[177.6, 22,737]	[-82%, 2174%]	0	0	0	0
	MPC	[177.6, 22,737]	[-82%, 2174%]	0.385	0.385	0	0
	Shuffle+MPC	[177.6, 22,737]	[-82%, 2174%]	0.385	0.385	0	0
HLL1	None	[277.9, 5,118]	[-72%, 412%]	0	0	0.04	0
	Shuffle	[277.9, 5,118]	[-72%, 412%]	0	0	0.03	0
	Rehash	[277.9, 5,118]	[-72%, 412%]	0	0.001	0	0.04
	Mask	[237.9, 5,118]	[-76%, 412%]	0	0	0	0
	MPC	[277.9, 5,118]	[-72%, 412%]	0.676	0.676	0	0
	Shuffle+MPC	[277.9, 5,118]	[-72%, 412%]	0.676	0.676	0	0
HLL4	None	[594.1, 1,498]	[-41%, 50%]	0.001	0.001	0.19	0
	Shuffle	[594.1, 1,498]	[-41%, 50%]	0.001	0.001	0	0
	Rehash	[594.1, 1,498]	[-41%, 50%]	0.001	0.002	0	0
	Mask	[594.1, 1,498]	[-41%, 50%]	0.001	0.001	0	0
	MPC	[594.1, 1,498]	[-41%, 50%]	4.764	4.764	0	0
	Shuffle+MPC	[594.1, 1,498]	[-41%, 50%]	4.764	4.764	0	0
HLL7	None	[839.9, 1,180]	[-16%, 18%]	0.006	0.006	1.67	2
	Shuffle	[839.9, 1,180]	[-16%, 18%]	0.006	0.006	0	2
	Rehash	[839.9, 1,180]	[-16%, 18%]	0.006	0.007	0	2
	Mask	[832.6, 1,251]	[-17%, 25%]	0.006	0.006	0	0
	MPC	[839.9, 1,180]	[-16%, 18%]	37.61	37.61	0	0
	Shuffle+MPC	[839.9, 1,180]	[-16%, 18%]	37.61	37.61	0	0
HLL15	None	[992.4, 1,008]	[-1%, 1%]	1.44	1.44	375	375
	Shuffle	[992.4, 1,008]	[-1%, 1%]	1.44	1.44	0	375
	Rehash	[992.4, 1,008]	[-1%, 1%]	1.601	1.621	0	375
	Mask	[93.02, 2,231]	[-91%, 123%]	0.267	0.267	0	0
Hashed IDs	None	[1000.0, 1000.0]	[0%, 0%]	0.002	0.002	1,919	1,919
	Rehash	[1000.0, 1000.0]	[0%, 0%]	0.002	0.002	0	1,919

Table A1e. 10,000 patient query.

Method	Obfuscation	Estimated number of patients		Wait (s)		Risk: Hub	Risk: Hub+Site
		Range of Counts	Relative Error	Mean	Max		
Count	None	[899.9, 19,470]	[-91%, 95%]	0	0	2.65	2.65
	Mask	[899.9, 19,477]	[-91%, 95%]	0	0	0	0
	MPC	[18,886, 19,470]	[89%, 95%]	0.099	0.099	0	0
HLL0	None	[1,421, 363,799]	[-86%, 3538%]	0	0	0.23	0.23
	Shuffle	[1,421, 363,799]	[-86%, 3538%]	0	0	0.23	0.23
	Rehash	[1,421, 363,799]	[-86%, 3538%]	0.001	0.01	0	0.23
	Mask	[1,421, 182,085]	[-86%, 1721%]	0	0	0	0
	MPC	[1,421, 363,799]	[-86%, 3538%]	0.384	0.384	0	0
	Shuffle+MPC	[1,421, 363,799]	[-86%, 3538%]	0.384	0.384	0	0
HLL1	None	[2,802, 56,273]	[-72%, 463%]	0	0	0.31	0.31
	Shuffle	[2,802, 56,273]	[-72%, 463%]	0	0	0.23	0.31
	Rehash	[2,802, 56,273]	[-72%, 463%]	0.001	0.01	0	0.31
	Mask	[2,559, 41,028]	[-74%, 310%]	0	0	0	0
	MPC	[2,802, 56,273]	[-72%, 463%]	0.676	0.676	0	0
	Shuffle+MPC	[2,802, 56,273]	[-72%, 463%]	0.676	0.676	0	0
HLL4	None	[6,243, 16,850]	[-38%, 68%]	0.001	0.001	1.84	1.84
	Shuffle	[6,243, 16,850]	[-38%, 68%]	0.001	0.001	0.23	1.84
	Rehash	[6,243, 16,850]	[-38%, 68%]	0.002	0.011	0	1.84
	Mask	[5,704, 16,369]	[-43%, 64%]	0.001	0.001	0	0
	MPC	[6,243, 16,850]	[-38%, 68%]	4.787	4.787	0.05	0.05
	Shuffle+MPC	[6,243, 16,850]	[-38%, 68%]	4.787	4.787	0	0.05
HLL7	None	[8,310, 11,347]	[-17%, 13%]	0.006	0.006	15.73	15.73
	Shuffle	[8,310, 11,347]	[-17%, 13%]	0.006	0.006	0.23	15.73
	Rehash	[8,310, 11,347]	[-17%, 13%]	0.007	0.016	0	15.73
	Mask	[7,167, 14,123]	[-28%, 41%]	0.005	0.005	0	0
	MPC	[8,310, 11,347]	[-17%, 13%]	37.83	37.83	0.3	0.3
	Shuffle+MPC	[8,310, 11,347]	[-17%, 13%]	37.83	37.83	0	0.3
HLL15	None	[9,928, 10,075]	[-1%, 1%]	1.462	1.462	3,707	3,707
	Shuffle	[9,928, 10,075]	[-1%, 1%]	1.462	1.462	0.23	3,707
	Rehash	[9,928, 10,075]	[-1%, 1%]	1.625	1.668	0	3,707
	Mask	[899.9, 19,477]	[-91%, 95%]	0.012	0.012	0	0
Hashed IDs	None	[10,000, 10,000]	[0%, 0%]	0.002	0.002	19,174	19,174
	Rehash	[10,000, 10,000]	[0%, 0%]	0.002	0.004	0	19,174

Table A1f. 100,000 patient query.

Method	Obfuscation	Estimated number of patients: Range of Counts	Estimated number of patients: Relative Error	Wait (s): Mean	Wait (s): Max	Risk: Hub	Risk: Hub+Site
Count	None	[9,057, 194,126]	[-91%, 94%]	0	0	0.02	0.02
	Mask	[9,057, 194,126]	[-91%, 94%]	0	0	0	0
	MPC	[188,850, 194,126]	[89%, 94%]	0.099	0.099	0	0
HLL0	None	[11,369, 727,598]	[-89%, 628%]	0	0	1.32	1.32
	Shuffle	[11,369, 727,598]	[-89%, 628%]	0	0	1.32	1.32
	Rehash	[11,369, 727,598]	[-89%, 628%]	0.011	0.096	0	1.32
	Mask	[11,369, 369,142]	[-89%, 269%]	0	0	0	0
	MPC	[11,369, 727,598]	[-89%, 628%]	0.385	0.385	0	0
	Shuffle+MPC	[11,369, 727,598]	[-89%, 628%]	0.385	0.385	0	0
HLL1	None	[20,470, 393,030]	[-80%, 293%]	0	0	2.71	2.71
	Shuffle	[20,470, 393,030]	[-80%, 293%]	0	0	1.32	2.71
	Rehash	[20,470, 393,030]	[-80%, 293%]	0.011	0.115	0	2.71
	Mask	[20,470, 226,929]	[-80%, 127%]	0	0	0	0
	MPC	[20,470, 393,030]	[-80%, 293%]	0.677	0.677	0	0
	Shuffle+MPC	[20,470, 393,030]	[-80%, 293%]	0.677	0.677	0	0
HLL4	None	[58,024, 169,675]	[-42%, 70%]	0.001	0.001	19.51	20
	Shuffle	[58,024, 169,675]	[-42%, 70%]	0.001	0.001	1.32	19.51
	Rehash	[58,024, 169,675]	[-42%, 70%]	0.012	0.123	0	19.51
	Mask	[45,325, 138,479]	[-55%, 38%]	0.001	0.001	0	0
	MPC	[58,024, 169,675]	[-42%, 70%]	4.771	4.771	0	0
	Shuffle+MPC	[58,024, 169,675]	[-42%, 70%]	4.771	4.771	0	0
HLL7	None	[79,547, 117,654]	[-20%, 18%]	0.006	0.006	158	158
	Shuffle	[79,547, 117,654]	[-20%, 18%]	0.006	0.006	1.32	158.3
	Rehash	[79,547, 117,654]	[-20%, 18%]	0.017	0.103	0	158
	Mask	[15,973, 190,297]	[-84%, 90%]	0.001	0.001	0	0
	MPC	[79,547, 117,654]	[-20%, 18%]	37.63	37.63	1.64	1.64
	Shuffle+MPC	[79,547, 117,654]	[-20%, 18%]	37.63	37.63	0	1.64
HLL15	None	[99,893, 101,691]	[-0%, 2%]	1.363	1.363	36,876	36,876
	Shuffle	[99,893, 101,691]	[-0%, 2%]	1.363	1.363	1	36,876
	Rehash	[99,893, 101,691]	[-0%, 2%]	1.537	1.637	0	36,876
	Mask	[9,057, 194,126]	[-91%, 94%]	0.001	0.001	0	0
Hashed IDs	None	[100,000, 100,000]	[0%, 0%]	0.008	0.008	191,759	191,759
	Rehash	[100,000, 100,000]	[0%, 0%]	0.011	0.032	0	191,759

Table A1g. 1,000,000 patient query.

Method	Obfuscation	Estimated number of patients		Wait (s)		Risk: Hub	Risk: Hub+Site
		Range of Counts	Relative Error	Mean	Max		
Count	None	[90,222, 1,940,247]	[-91%, 94%]	0	0	0	0
	Mask	[90,222, 1,940,247]	[-91%, 94%]	0	0	0	0
	MPC	[1,888,932, 1,940,247]	[89%, 94%]	0.1	0.1	0	0
HLL0	None	[181,899, 17,753,385]	[-82%, 1675%]	0	0	11.71	11.71
	Shuffle	[181,899, 17,753,385]	[-82%, 1675%]	0	0	11.71	11.71
	Rehash	[181,899, 17,753,385]	[-82%, 1675%]	0.104	0.947	0	11.71
	Mask	[90,950, 932,550]	[-91%, -7%]	0	0	0	0
	MPC	[181,899, 17,753,385]	[-82%, 1675%]	0.385	0.385	0.2	0.2
	Shuffle+MPC	[181,899, 17,753,385]	[-82%, 1675%]	0.385	0.385	0.2	0.2
HLL1	None	[206,887, 11,581,296]	[-79%, 1058%]	0	0	23.06	23.06
	Shuffle	[206,887, 11,581,296]	[-79%, 1058%]	0	0	12.15	23.06
	Rehash	[206,887, 11,581,296]	[-79%, 1058%]	0.105	0.947	0	23.06
	Mask	[109,175, 1,273,729]	[-89%, 27%]	0	0	0	0
	MPC	[206,887, 11,581,296]	[-79%, 1058%]	0.679	0.679	0.39	0.39
	Shuffle+MPC	[206,887, 11,581,296]	[-79%, 1058%]	0.679	0.679	0.2	0.39
HLL4	None	[538,869, 1,713,823]	[-46%, 71%]	0.001	0.001	186.3	186.3
	Shuffle	[538,869, 1,713,823]	[-46%, 71%]	0.001	0.001	12.41	186.3
	Rehash	[538,869, 1,713,823]	[-46%, 71%]	0.105	0.943	0	186.3
	Mask	[90,644, 1,892,176]	[-91%, 89%]	0	0	0	0
	MPC	[538,869, 1,713,823]	[-46%, 71%]	4.754	4.754	1.91	1.91
	Shuffle+MPC	[538,869, 1,713,823]	[-46%, 71%]	4.754	4.754	0.2	1.91
HLL7	None	[837,269, 1,158,432]	[-16%, 16%]	0.006	0.006	1,508	1,508
	Shuffle	[837,269, 1,158,432]	[-16%, 16%]	0.006	0.006	12.48	1,508
	Rehash	[837,269, 1,158,432]	[-16%, 16%]	0.111	0.955	0	1,508
	Mask	[90,222, 1,940,247]	[-91%, 94%]	0.001	0.001	0	0
	MPC	[837,269, 1,158,432]	[-16%, 16%]	37.39	37.39	14.71	14.71
	Shuffle+MPC	[837,269, 1,158,432]	[-16%, 16%]	37.39	37.39	0.2	14.71
HLL15	None	[987,401, 1,011,968]	[-1%, 1%]	1.379	1.379	348,258	348,258
	Shuffle	[987,401, 1,011,968]	[-1%, 1%]	1.379	1.379	12.5	348,258
	Rehash	[987,401, 1,011,968]	[-1%, 1%]	1.652	2.537	0	348,258
	Mask	[90,222, 1,940,247]	[-91%, 94%]	0.001	0.001	0	0
Hashed IDs	None	[1,000,000, 1,000,000]	[0%, 0%]	0.071	0.071	1,917,817	1,917,817
	Rehash	[1,000,000, 1,000,000]	[0%, 0%]	0.097	0.302	0	1,917,817

Table A1h. 10,000,000 patient query.

Method	Obfuscation	Estimated number of patients		Wait (s)		Risk: Hub	Risk: Hub+Site
		Range of Counts	Relative Error	Mean	Max		
Count	None	[902,793, 19,402,938]	[-91%, 94%]	0	0	0	0
	Mask	[902,793, 19,402,938]	[-91%, 94%]	0	0	0	0
	MPC	[18,884,586, 19,402,938]	[89%, 94%]	0.105	0.105	0	0
HLL0	None	[1,455,196, 71,013,541]	[-85%, 610%]	0	0	70.06	70
	Shuffle	[1,455,196, 71,013,541]	[-85%, 610%]	0	0	70	70
	Rehash	[1,455,196, 71,013,541]	[-85%, 610%]	1.032	9.685	0	70
	Mask	[734,987, 17,239,998]	[-93%, 72%]	0	0	0	0
	MPC	[1,455,196, 71,013,541]	[-85%, 610%]	0.383	0.383	1	1
	Shuffle+MPC	[1,455,196, 71,013,541]	[-85%, 610%]	0.383	0.383	1	1
HLL1	None	[2,215,613, 41,923,242]	[-78%, 319%]	0	0	140.8	141
	Shuffle	[2,215,613, 41,923,242]	[-78%, 319%]	0	0	92.64	140.8
	Rehash	[2,215,613, 41,923,242]	[-78%, 319%]	1.033	9.483	0	141
	Mask	[887,447, 18,931,827]	[-91%, 89%]	0	0	0	0
	MPC	[2,215,613, 41,923,242]	[-78%, 319%]	0.675	0.675	2	2
	Shuffle+MPC	[2,215,613, 41,923,242]	[-78%, 319%]	0.675	0.675	1.17	2
HLL4	None	[6,171,672, 16,428,565]	[-38%, 64%]	0.001	0.001	1,123	1,123
	Shuffle	[6,171,672, 16,428,565]	[-38%, 64%]	0.001	0.001	122	1,123
	Rehash	[6,171,672, 16,428,565]	[-38%, 64%]	1.035	9.495	0	1,123
	Mask	[902,793, 19,402,938]	[-91%, 94%]	0.001	0.001	0	0
	MPC	[6,171,672, 16,428,565]	[-38%, 64%]	4.755	4.755	18	18
	Shuffle+MPC	[6,171,672, 16,428,565]	[-38%, 64%]	4.755	4.755	1.18	18
HLL7	None	[8,352,290, 12,307,282]	[-16%, 23%]	0.006	0.006	8,947	8,947
	Shuffle	[8,352,290, 12,307,282]	[-16%, 23%]	0.006	0.006	126	8,947
	Rehash	[8,352,290, 12,307,282]	[-16%, 23%]	1.043	9.496	0	8,947
	Mask	[902,793, 19,402,938]	[-91%, 94%]	0.001	0.001	0	0
	MPC	[8,352,290, 12,307,282]	[-16%, 23%]	37.41	37.41	144	144
	Shuffle+MPC	[8,352,290, 12,307,282]	[-16%, 23%]	37.41	37.41	1.18	144
HLL15	None	[9,887,923, 10,123,720]	[-1%, 1%]	1.392	1.392	2,127,583	2,127,583
	Shuffle	[9,887,923, 10,123,720]	[-1%, 1%]	1.392	1.392	127	2,127,583
	Rehash	[9,887,923, 10,123,720]	[-1%, 1%]	2.641	11.55	0	2,127,583
	Mask	[902,793, 19,402,938]	[-91%, 94%]	0.001	0.001	0	0
Hashed IDs	None	[10,000,000, 10,000,000]	[0%, 0%]	0.794	0.794	19,178,114	19,178,114
	Rehash	[10,000,000, 10,000,000]	[0%, 0%]	1.045	3.09	0	19,178,114

Table A1i. 100,000,000 patient query.

Method	Obfuscation	Estimated number of patients		Wait (s)		Risk: Hub	Risk: Hub+Site
		Range of Counts	Relative Error	Mean	Max		
Count	None	[9,431,902, 200,018,431]	[-91%, 100%]	0	0	0	0
	Mask	[9,431,902, 200,018,431]	[-91%, 100%]	0	0	0	0
	MPC	[199,984,637, 200,018,431]	[100%, 100%]	0.171	0.171	0	0
HLL0	None	[11,641,564, 818,287,824]	[-88%, 718%]	0	0	99.97	99.97
	Shuffle	[11,641,564, 818,287,824]	[-88%, 718%]	0	0	99.97	99.97
	Rehash	[11,641,564, 818,287,824]	[-88%, 718%]	9.42	84.36	0	99.97
	Mask	[9,431,902, 200,018,431]	[-91%, 100%]	0.001	0.001	0	0
	MPC	[11,641,564, 818,287,824]	[-88%, 718%]	0.387	0.387	9.91	9.91
	Shuffle+MPC	[11,641,564, 818,287,824]	[-88%, 718%]	0.387	0.387	9.91	9.91
HLL1	None	[22,952,975, 448,641,535]	[-77%, 349%]	0	0	199.9	199.9
	Shuffle	[22,952,975, 448,641,535]	[-77%, 349%]	0	0	198.5	199.9
	Rehash	[22,952,975, 448,641,535]	[-77%, 349%]	9.42	84.36	0	199.9
	Mask	[9,431,902, 200,018,431]	[-91%, 100%]	0.001	0.001	0	0
	MPC	[22,952,975, 448,641,535]	[-77%, 349%]	0.681	0.681	19.82	19.82
	Shuffle+MPC	[22,952,975, 448,641,535]	[-77%, 349%]	0.681	0.681	10.75	19.82
HLL4	None	[57,717,266, 167,663,627]	[-42%, 68%]	0.001	0.001	1,599	1,599
	Shuffle	[57,717,266, 167,663,627]	[-42%, 68%]	0.001	0.001	854.1	1,599
	Rehash	[57,717,266, 167,663,627]	[-42%, 68%]	9.422	84.36	0	1,599
	Mask	[9,431,902, 200,018,431]	[-91%, 100%]	0.001	0.001	0	0
	MPC	[57,717,266, 167,663,627]	[-42%, 68%]	4.768	4.768	153.8	153.8
	Shuffle+MPC	[57,717,266, 167,663,627]	[-42%, 68%]	4.768	4.768	11.69	153.8
HLL7	None	[84,484,568, 117,231,365]	[-16%, 17%]	0.006	0.006	12,795	12,795
	Shuffle	[84,484,568, 117,231,365]	[-16%, 17%]	0.006	0.006	1,199	12,795
	Rehash	[84,484,568, 117,231,365]	[-16%, 17%]	9.426	84.36	0	12,795
	Mask	[9,431,902, 200,018,431]	[-91%, 100%]	0.001	0.001	0	0
	MPC	[84,484,568, 117,231,365]	[-16%, 17%]	37.43	37.43	1,246	1,246
	Shuffle+MPC	[84,484,568, 117,231,365]	[-16%, 17%]	37.43	37.43	11.83	1,246
HLL15	None	[99,097,598, 101,037,154]	[-1%, 1%]	1.362	1.362	3,259,256	3,259,256
	Shuffle	[99,097,598, 101,037,154]	[-1%, 1%]	1.362	1.362	1,265	3,259,256
	Rehash	[99,097,598, 101,037,154]	[-1%, 1%]	10.96	85.89	0	3,259,256
	Mask	[9,431,902, 200,018,431]	[-91%, 100%]	0.001	0.001	0	0
Hashed IDs	None	[100,000,000, 100,000,000]	[0%, 0%]	10.12	10.12	199,999,801	199,999,801
	Rehash	[100,000,000, 100,000,000]	[0%, 0%]	11.56	23.31	0	199,999,801
