# Supplementary material for: Balancing Accuracy and Privacy in Federated Queries of Clinical Data Repositories: Algorithm Development and Validation
Source: J Med Internet Res. 2020 Nov 3;22(11):e18735. doi: 10.2196/18735 (PMC7671849; doi:10.2196/18735)
Supplement: Multimedia Appendix 3 [file jmir_v22i11e18735_app3.pdf]

Multimedia Appendix 3

Federated queries of clinical data repositories: balancing accuracy and privacy

Yun William Yu, PhD
Computer and Mathematical Sciences
University of Toronto at Scarborough
Toronto, ON M5S 1J7
ywyu@math.toronto.edu

Griffin M Weber, MD, PhD
Department of Biomedical Informatics
Harvard Medical School
Boston, MA 02115
weber@hms.harvard.edu

Table A2. Time and space complexity for various methods

Theoretical upper bounds and actual added compute time when using a particular method + obfuscation. Also shown is the space-complexity (both theoretical and empirical) of the amount of data that the hospitals and hub have to send over the network. Note that actual times given are based on assuming that the hospitals run their local computations in parallel. Further, note that while we have only given asymptotic theoretical time-complexities in big-O notation, the theoretical communication complexity (Transfer Time – Bytes) is an exact number based on the parameters. In the table, *n*=number of hospitals, *m*=patients per hospital, and *t*=number of HLL buckets.

**Algorithm**		**Added time complexity**			**Actual additional time (s)**		**Actual time examples (s)**		**Transfer time (theoretical)**		**Actual data**
**Method**	**Obfuscation**	**Sketch**	**Obfuscate**	**Hub**	**Site**	**Hub**	**1 Patient**	**100M Patients**	**Rounds**	**Bytes**	**100M Patients**
Count				O(n)		$10^{-7}n$	0.000	0.000	1	$4n$	400 bytes
Count	Mask		O(1)	O(n)	10^-7	$10^{-7}n$	0.000	0.000	1	$4n$	400 bytes
Count	MPC		O(1)	O(n)	0.049	$0.05 + 0.006n$	0.100	0.100	3	$896n$	87.5 KiB
HLL		O(t)		O(nt)		$5 \times 10^{-9} \cdot nt$	0.006	0.005	1	nt	12800 bytes
HLL	Mask	O(t)	O(t)	O(nt)	$10^{-7} \cdot t$	$5 \times 10^{-9} \cdot nt$	0.007	0.001	1	nt	12800 bytes
HLL	Shuffle	O(t)	O(t)	O(nt)	$10^{-7} \cdot t$	$5 \times 10^{-9} \cdot nt$	0.006	0.005	1	nt	12800 bytes
HLL	Rehash	O(t)	O(m)	O(nt)	$6.5 \times 10^{-6} \cdot m$	$5 \times 10^{-9} \cdot nt$	0.007	8.757	1	nt	12800 bytes
HLL	MPC	O(t)	O(t)	O(nt)	$0.05t$	$0.00235nt + 0.197t + 0.027$	40.930	41.010	3	$256n + 20480nt$	250 MiB
HLL	Shuffle+MPC	O(t)	O(t)	O(nt)	$0.05t$	$0.00235nt + 0.197t + 0.027$	40.930	41.010	3	$256n + 20480nt$	250 MiB
HashedIDs				O(mn)		$10^{-7} \cdot mn$	0.003	12.670	1	$4m$	8 MB
HashedIDs	Rehash		O(m)	O(mn)	$6.5 \times 10^{-6} \cdot m$	$10^{-7} \cdot mn$	0.003	14.350	1	$4m$	8 MB
